# Mosaic loss of chromosome Y in blood is associated with male susceptibility for idiopathic pulmonary fibrosis

**DOI:** 10.1038/s43856-025-00966-9

**Published:** 2025-06-28

**Authors:** Josefin Bjurling, Nicholas W. Chavkin, Jonatan Halvardson, Mark C. Thel, Jonas Mattisson, John S. Kim, Ammar Zaghlool, Shwu-Fan Ma, Fernando J. Martinez, Kevin Anstrom, Imre Noth, Kenneth Walsh, Lars A. Forsberg

**Affiliations:** 1 Department of Immunology, Genetics and Pathology, Science for Life Laboratory, Uppsala University, Uppsala, Sweden; 2 Hematovascular Biology Center, Robert M. Berne Cardiovascular Research Center, University of Virginia School of Medicine, Charlottesville, VA USA; 3 Center for Developmental Biology and Regenerative Medicine, Seattle Children’s Research Institute, Seattle, WA USA; 4 Department of Pediatrics, University of Washington School of Medicine, Seattle, WA USA; 5 Division of Pulmonary & Critical Care Medicine, University of Virginia School of Medicine, Charlottesville, VA USA; 6 Department of Medicine, University of Virginia School of Medicine, Charlottesville, VA USA; 7 Department of Medicine, Weill Cornell Medical Center, New York, NY USA; 8 University of North Carolina at Chapel Hill Gillings School of Global Public Health, Chapel Hill, NC USA

**Keywords:** Respiratory tract diseases, Predictive markers

## Abstract

**Background:**

The prevalence of idiopathic pulmonary fibrosis (IPF) is higher in men, a previously not well-understood sex bias that extends across severity and mortality. Mosaic loss of chromosome Y (mLOY) in blood is male-specific and associated with adverse outcomes, including IPF. In mLOY-mice, enhanced TGF-β signaling has been reported to contribute to fibrosis of internal organs, but it is not known if such mLOY-driven disease mechanism exists in humans.

**Methods:**

We focused here on IPF in men to investigate if mLOY contributes to fibrotic disease processes in humans, as demonstrated in mouse models. To this end, we investigated mLOY as a risk factor for male IPF in epidemiological and clinical datasets, as well as by re-analyses of published single-cell RNA sequencing (scRNAseq) datasets.

**Results:**

We find that men with mLOY in blood display an increased risk for IPF diagnosis and death caused by IPF in UK Biobank, and that mLOY is associated with reduced lung functions in two cohorts. Approximately 80% of the male excess in IPF prevalence occurs in the group of men with mLOY in blood leukocytes. Notably, scRNAseq analyses support that pulmonary leukocytes with Y loss exacerbate IPF by upregulating profibrotic genes and enhancing TGF-β signaling.

**Conclusions:**

Our results contribute to explaining the profound sex bias in IPF and replicate a mLOY-driven profibrotic disease mechanism first identified in mice. Male IPF patients with mLOY represent a subgroup that may benefit from treatment with TGF-β inhibitors.

## Plain language summary

Idiopathic pulmonary fibrosis (IPF) is a lung disease that affects more men than women, but the reason for this sex difference has been unclear. Here we show that a male-specific genetic change, called mosaic loss of chromosome Y (mLOY), increases the risk of IPF in men. We analyzed large health databases and genetic data, finding that men with mLOY in their blood had a higher risk of developing IPF, worse lung function, and were more likely to die from the disease. Studies in mice have shown that when some white blood cells lose the Y chromosome, it can lead to increased scarring (fibrosis) in the lungs and other internal organs. In this study, we show that the same process can also happen in men. These findings help explain why IPF is more common in men and suggest that men with mLOY could benefit from targeted treatments.

## Abstract (web summary)

Bjurling et al. evaluate mosaic loss of chromosome Y (mLOY) as a risk factor for male idiopathic pulmonary fibrosis (IPF) in epidemiological, clinical, and single-cell RNA sequencing data. The results suggest that mLOY contributes to IPF severity by enhancing profibrotic signaling in lung tissues, highlighting the potential for targeted therapies.

## Introduction

Idiopathic pulmonary fibrosis (IPF) is a chronic disease characterized by excessive accumulation of fibrotic extracellular matrix (ECM)^1^. The prevalence of IPF varies between populations ranging from 0.33 to 2.98 per 10,000 individuals^2^. Although approved antifibrotic treatment slows disease progression, the overall prognosis is poor (median survival: 3–4 years from diagnosis) and effective cures are missing^1,2^. Known risk factors include male sex, older age, smoking, environmental exposures and genomic variants^1,3,4^. The influence of sex in IPF extends across incidence, disease severity, and mortality, but the cause of male vulnerability for IPF has been largely unknown^3,5^.

Mosaic loss of chromosome Y (mLOY) is a male-specific somatic mutation that accumulates in the hematopoietic system of aging men and is associated with increased risk for all-cause mortality^6–9^. Risk factors for mLOY include increased age, smoking, genetic predisposition, and environmental exposures^7,10–12^. Analysis of bulk DNA samples shows that at least 20% overall and up to 40% of 70-year-old UK Biobank (UKB) men are carriers of hematological immunocytes without the Y chromosome^10^. Furthermore, studies of chromosome Y loss in single cells suggest that all aging men might be affected^10,13,14^ and that Y loss occurs in multiple blood cell progenitors during life^15^. In addition to its high prevalence, hematopoietic Y loss is associated with increased risk for all major causes of human death, including cardiovascular diseases, cancer, and Alzheimer’s disease^6–8,16–18^. Recently, results from two large prospective cohorts describe mLOY in blood leukocytes to be associated with increased risk of incident lung diseases, including IPF^19^, a result that did not, however, replicate in a smaller Mexican cohort^20^. The mechanism(s) of how mLOY in blood might increase the risk for disease in other organs is currently being investigated, and recently, enhanced organ fibrosis, as a direct consequence of induced hematological Y loss, has been reported in mouse models^8^. Fibrosis accounts for substantial morbidity and is a pathologic feature of various diseases affecting virtually all organs, including the lungs in IPF^21^.

In this study, we show that men with mLOY in blood have a higher risk of being diagnosed with, and dying from, IPF in the UK Biobank. We also find that mLOY in blood is linked to reduced lung function in two independent cohorts. We observe that about 80% of the male excess in IPF prevalence occurs in the group of men with mLOY in blood leukocytes. Furthermore, re-analysis of published single-cell RNA sequencing (scRNAseq) datasets indicates that mLOY in leukocytes contributes to fibrotic disease processes in humans, consistent with findings from mouse models.

## Methods

### IPF and lung function in relation to hematological mLOY in UKB

The UKB is a prospective cohort encompassing genetic, lifestyle, health, and outcomes information from half a million UK participants, 40–70 years old at study entry^22^. UKB is approved by the North West Multi-Centre Research Ethics Committee (Ref. 11/NW/0382) and all participants provided written informed consent to participate. The present study was conducted under application number 61272 using a dataset extracted on February 7, 2024, which was used as the time of censoring, giving a median follow-up time of 14.9 years. Incidence and mortality from IPF (ICD10 code J84.1 in the 10th revision of the International Statistical Classification of Diseases and Related Health Problems) were assessed as registered in the UKB database. IPF outcomes registered as the main or contributory diagnosis or cause of death were included. Investigation of incidence includes both prevalent and incident cases. Covariates used were extracted from UK Biobank data-fields 21003 (age), 22009 (genetic principal components), and 20116 (smoking habits). Packyears was constructed using data-field 20161 and packyears for non-smokers which were not recorded as previous smokers in data-field 20116 were set to zero. A vector for alcohol consumption was constructed by combining data-fields 20117 (alcohol drinker status) and 1558 (alcohol intake frequency). Data-fields 24003 (Nitrogendioxide), 24004 (NitrogenOxides), 24005 (particulate matter (PM) 10), 24007 (PM2.5), and 24008 (PM2.5-PM10) were used for environmental exposures. Missing values in environmental exposures were imputed using the median. Forced vital capacity (FVC) (data-field 3062) from baseline assessment was investigated in relation to mLOY for men with acceptable spirometry measures (data-field 20152).

### Assessment of hematological mLOY in UKB participants

The percentage of leukocytes with Y loss was calculated for all male participants using intensity data from SNP-array genotyping of blood samples collected at study entry, as before^7,8^. First, the median log *R* values of probes specific to the X and Y chromosomes were calculated for all participants self-reported as male, i.e., mLRRX and mLRRY. These estimates are indicative of mosaic chromosomal aberrations and were inspected to exclude genetic females and male participants with sex chromosome abnormalities other than mLOY, such as 47XYY and 47XXY subjects (Supplementary Fig. 1). The final dataset was further curated by the removal of samples with low genotyping quality as defined by the derivative log-ratio spread (DLRS) using a threshold of 1.5 interquartile ranges from the third quartile of the DLRS distribution, i.e., DLRS values above 0.35 for chromosome 1 probes. Finally, all subjects expressing a wish to withdraw their participation were excluded, leaving a dataset consisting of 216,608 male participants. Individual mLRRY values were next adjusted for batch effects and transformed into a percentage of leukocytes with Y loss in each male participant using previously validated methods^23,24^. Thresholds representing Y loss in at least 8.28% (as further described in “Statistical analyses” below) or 40% of blood leukocytes were applied for categorical scoring of mLOY with low (but robustly detectable) or high levels of Y loss, respectively^7^.

### Lung function in relation to hematological mLOY in CleanUP-IPF

Clinical Efficacy of Antimicrobial Therapy Strategy Using Pragmatic Design in Idiopathic Pulmonary Fibrosis (CleanUP-IPF, NCT02759120)^25^ was a clinical trial that enrolled a total of 513 IPF patients across 35 US sites with a mean age of 71 years and 78.4% of whom were men, who were included in this analysis. The study was approved by institutional review boards at each institution, and all participants provided written informed consent. We investigated whether mLOY in male IPF patients was associated with two measures of lung function, FVC and diffusing capacity of carbon monoxide (DLCO)^26^. CleanUP-IPF was approved by institutional review boards at each institution, and each patient provided written informed consent^25^.

### Assessment of hematological mLOY in CleanUP-IPF

Genomic DNA (gDNA) was isolated from baseline peripheral blood samples obtained from 400 IPF patients using PAXgene Blood DNA kit (Cat no. 761133, Qiagen). Extracted gDNA was diluted to 15 ng/μL and stored at −80 °C until use. Peripheral blood mLOY was measured using digital PCR^23^. A Taqman assay (Cat no. C_990000001_10, Thermo Fisher Scientific) was employed to estimate the relative number of copies of *AMELY* and *AMELX*, serving as proxies for the number of Y and X chromosomes, respectively. Separate FAM and VIC-tagged probes were used to target a 6-bp difference between *AMELY* and *AMELX*. Samples of gDNA were mixed with QIAcuity CNV Probe Mastermix (Cat no. 250102, Qiagen) and the Taqman assay primer/probes. Solutions were then partitioned using the Qiagen 26k Nanoplates (Cat no. 250001, Qiagen) on the QIAcuity One 2Plex system (Cat no. 911001, Qiagen). The manufacturer’s recommendation for amplification was followed: 95 °C for 2 min, 40 cycles 95 °C for 15 s, and 60 °C for 30 s. After thermocycling, the plates were imaged and the estimated concentration of *AMELY* and *AMELX* was determined from the relative amount of FAM and VIC fluorescence with the QIAcuity One Software Suite (v 2.2.0.26). The ratio of *AMELY*/*AMELX* in each sample was extended to calculate mLOY using the following equation (1 − [*AMELY*]/[*AMELX*]) × 100%. A threshold of mLOY in 40% of blood leukocytes was applied to assess if high levels of mLOY in blood have a potential impact on lung function in IPF patients.

### LOY in pulmonary tissues

The occurrence of LOY-cells in pulmonary tissues and transcriptional effects in pulmonary LOY-leukocytes were explored by re-analyzing five publicly available scRNAseq datasets generated from pulmonary tissues from IPF patients and controls, i.e., Morse et al. (GSE128033)^27^, Reyfman et al. (GSE122960)^28^, Adams et al. (GSE136831)^29^, Habermann et al. (GSE135893)^30^, and de Rooij et al. (GSE159585)^31^. Each study has ethical approval as described therein. For investigation of the frequency of Y loss in different types of cells resident in lung tissues, the Adams et al. dataset was used since this dataset had the best age-matching between patients and control donors.

### Characterization of Y loss in pulmonary single cells from IPF patients

The scRNAseq dataset from Adams et al.^29^ consists of samples from IPF patients, chronic obstructive pulmonary disease (COPD) patients and control donor lungs. The analysis was carried out in R (v 4.2.2). Females and individuals with COPD were discarded from the dataset of 312,928 cells. The remaining 161,011 cells were processed using Seurat (v 4.3.0). After removing low-quality cells, with >10% mitochondrial DNA and <800 and >3500 expressed genes, 88,466 single cells remained. Data normalization and scaling were performed according to the standard execution in Seurat followed by calculations of principal components (PCs) using the 2000 most variable features. Interindividual effects in gene expression were removed using Harmony (v 0.1.1). Cell clusters were constructed using the 31 first PCs with 0.5 resolution. To assign which cell type each cluster represented, four genes with the highest differential expression compared to other clusters were used. The list of genes was manually curated using the tissue and single-cell expression profiles from the human protein atlas (HPA) (www.proteinatlas.org)^32^. The assigned cell types were visualized using Uniform Manifold Approximation and Projection (UMAP)^33^. To improve the resolution of subpopulations, subclusters were constructed using the first 31 PCs and 0.4 resolution, and assigned using HPA profiles. Macrophages were further annotated based on the expression of the following genes: M1 macrophages by *IL1B*, M2 macrophages by *MRC1*, M2a by *FN1* and *TGM2*, M2b by *IL1B*, M2c by *MERTK*, and M2d by *VEGFA* (Supplementary Fig. 2)^34–36^. Next, Y chromosome loss in a single cell was defined as a lack of expression of genes located in the male-specific region of chromosome Y (MSY)^10,14^. Genes located in MSY, identified using Ensembl (release 91), were extracted from the gene expression data. The expression across all MSY genes was estimated for each cell by summarizing the number of transcripts mapped to these genes, and cells with complete absence of MSY gene expression were classified as LOY-cells. To investigate Y loss in IPF patients, we pooled scRNAseq data from 26 IPF cases (age range = 54–78 years) generating a set of 57,707 cells. As a control group, we pooled data from six participants without IPF in a similar age range (50–65 years), comprising 10,792 cells.

### Investigation of single-cell data for functional effects of Y loss

To examine LOY-associated cellular effects in single cells, four additional published scRNAseq datasets were included, i.e., Morse et al.^27^, Reyfman et al.^28^, Habermann et al.^30^, and de Rooij et al.^31^. Single cells from men with >4000 reads were included in analyses of gene expression using Seurat (v 4.0). Dataset integration was performed according to the SCTIntegration pipeline. LOY-cells were identified as described above. Cell clusters were annotated based on gene expression: leukocytes by *PTPRC*, endothelial cells by *CDH5*, epithelial cells by *EPCAM*, fibroblasts by *PDGFRA*, and vascular smooth muscle cells by *ACTA2* (Supplementary Fig. 3a). Within the leukocyte subset, cells were re-clustered and annotated based on gene expression: macrophages by *CD68*, monocytes by *CSF1R*, neutrophils by *CSF3R*, T cells by *CD3G*, B cells by *CD79A*, NK cells by *NCAM1*, and dendritic cells by *CD80* (Supplementary Fig. 3b). Cells were defined by cluster classifications described above, and leukocytes were divided into wild-type (WT) cells and LOY-cells to determine LOY-mediated differences in interactions. The probability of interactions in given cell signaling pathways was determined. The analysis of differential gene expression (DGE) was performed using Seurat (v 4.3.0). Enrichment of Gene Ontology (GO) terms was performed using the Gene Ontology Consortium^37^. Next, interactions between cell types within healthy, non-IPF (COPD/COVID), or IPF patients were determined by analysis using CellChat (v 1.6.1) within R. Standard input parameters in the protocol, provided by the original authors, were used to quantify WT and LOY leukocyte interactions with other annotated cell types and compare the strength of signaling pathways. Fibroblast activation score in each patient was determined by the average expression of genes from patient-derived cells previously associated with activated fibroblast populations in lung fibrosis^38,39^ as quantified by the AddModuleScore function in Seurat.

### Statistical analyses

All statistical analyses of UKB data were carried out using R (v 4.3.1). Fisher’s exact test was carried out using fisher.test (v 4.3.1). Logistic regression was performed using the R glm function (v 4.3.1), and Cox proportional hazards regression models were performed using the survival package (v 3.5.5) and the coxph function. In statistical analyses, mLOY was considered as a continuous variable and as a categorical variable. For categorical scoring of men with or without Y loss in UKB, a statistically derived threshold based on the lower limit of the 99% confidence interval of the experimental noise of the genotyping data was used^7^, representing Y loss in at least 8.28% of blood leukocytes in the current dataset. Mediation analysis was performed using the R mediate package (v 4.5.0). A linear regression model with age and the five first genetic PCs as covariates was used as the model.m argument and the same logistic regression reported as the full analysis was used as the model.y argument. In CleanUP-IPF, men with high and low levels of mLOY were defined using a threshold of 40% (Supplementary Fig. 4). FVC and DLCO in CleanUP-IPF were investigated in relation to mLOY using linear regression adjusting for baseline confounders. The level of LOY-cells in lung resident cell types was compared between cases and controls in the Adams et al. scRNAseq dataset using the fisher.test function from the stats package (v 4.2.2) in R. Statistical analyses to investigate the functional effects of Y loss in the combined scRNAseq dataset were performed by internal software analyses and presented as provided.

## Results

### Chromosome Y loss in blood leukocytes is associated with the risk for IPF

**Fig. 1:**
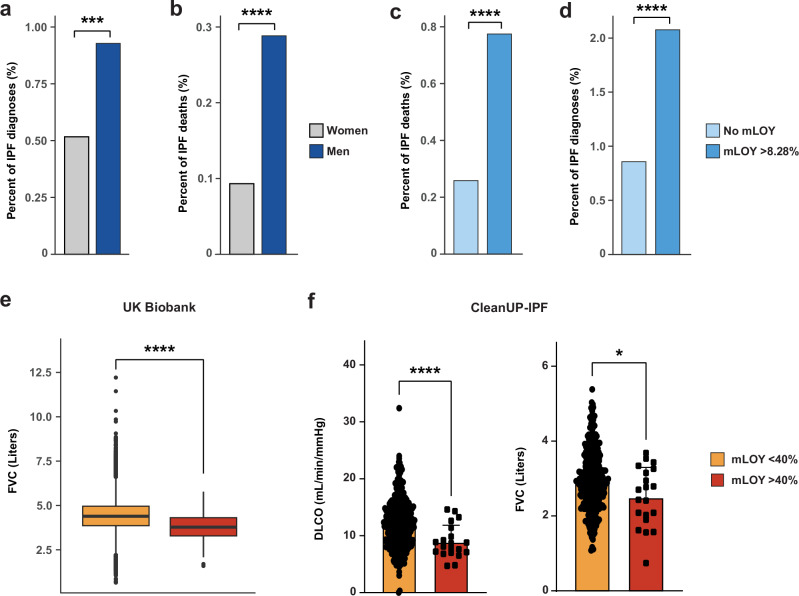
Hematopoietic Y loss is associated with IPF and reduced lung function. **a**–**d** Incidence and mortality from idiopathic pulmonary fibrosis (IPF) in relation to sex, age, and mosaic loss of chromosome Y (mLOY) in UKB participants after a median follow-up time of 14.9 years, using a threshold for mLOY scoring at 8.28%. The frequency of registered IPF diagnoses among male and female participants (**a**) and men with or without mLOY (**d**). **b** shows the percentage of deaths caused by IPF in men and women, while **c** shows corresponding percentages in men with or without mLOY. **e**, **f** show the association between mLOY in blood and lung function, using a threshold for mLOY scoring at 40%. **e** Forced vital capacity (FVC) measured at the initial assessment visit was compared between men based on mLOY status in UKB. Error bars represent the 95% confidence interval. **f** FVC and diffusing capacity of carbon monoxide (DLCO) in relation to mLOY in CleanUP-IPF. Significant differences are denoted as: ****p < 0.0001, ***p < 0.001, *p < 0.05.

We first investigated IPF in relation to mLOY in the prospective UKB cohort with participants from the general population. Among 407,034 participants included in the present analysis, 2845 men and women were diagnosed with IPF before or after study entry, while 731 subjects died from IPF during a median follow-up time of 14.9 years. Our initial unadjusted analyses confirmed previously known sex-bias in IPF, with higher incidence and mortality from IPF among men compared with women (Fig. 1a, b, Supplementary Fig. 5, and Supplementary Table 1). The level of mLOY in IPF patients and controls in relation to age is shown in Supplementary Fig. 6.

To examine hematological Y loss as a risk factor for IPF, we first investigated IPF mortality during follow-up among UKB men with mLOY in blood at study entry using multivariable-adjusted Cox proportional hazards regression. The percent of mLOY in blood leukocytes at baseline was used as a continuous dependent variable while adjusting the risk estimate for confounders such as age, smoking, environmental exposures and other relevant risk factors (Supplementary Table 2). Follow-up times were calculated from the date of blood draw until the date of death or censoring. This adjusted survival analysis showed that increased level of mLOY in blood at baseline was associated with greater risk to die from IPF during the follow-up time (hazard ratio = 1.013 per percent increase of mLOY, 95% confidence interval (CI) = 1.003–1.022, *p* = 0.009, *n* = 520, Supplementary Table 3). In addition to validating mLOY as a risk factor for IPF death, this analysis replicated previously known risk factors such as age, smoking and exposure to nanoparticles. Next, a categorical LOY-variable was constructed to score individuals with or without detectable mLOY at baseline using a threshold representing Y loss in at least 8.28% of leukocytes in the present dataset. Investigating IPF mortality in the two groups showed that the absolute risk for IPF death during follow-up was 0.77% and 0.26% among men scored with or without Y loss at baseline, respectively (Fig. 1c). In contrast, only 0.09% of the women in UKB had died from IPF at the date of data collection. The relative risk for death in IPF among men with hematopoietic Y loss was next estimated using the same categorical mLOY-variable in a multivariable Cox model adjusting for the same baseline confounders as in the primary survival analysis. This analysis showed that the group of men who scored with Y loss at study entry exhibited a 33.7% higher risk to die from IPF during follow-up compared with other men (Supplementary Table 4).

The incidence of IPF among UKB men with mLOY in blood was next analyzed using a logistic regression model adjusted for the same baseline confounders as in the survival models described above, including age, smoking and environmental exposures (Supplementary Table 2). IPF diagnosis registered before or after baseline was used as the response variable and Y loss was first modeled as a continuous variable. The adjusted risk estimate from this analysis shows that a greater level of mLOY in blood leukocytes was associated with increased risk for IPF diagnosis (odds ratio (OR) = 1.01 per percent increase of mLOY, CI = 1.004–1.016, *p* = 0.0004, *n* = 1673, Supplementary Table 5). Investigating absolute risks for IPF diagnosis showed that 2.07% of men who scored with Y loss had been diagnosed with IPF at the date of data collection (Fig. 1d), compared to 0.86% of other men and 0.52% of women. Adjusted logistic regression using the categorical mLOY-variable shows that the relative risk for IPF diagnosis was 17.3% higher in the group of men scored with Y loss compared with other men (Supplementary Table 6).

Next, we explored the UKB data to estimate the part of the observed sex difference in IPF that could potentially be attributed to mLOY. This was performed by investigating male excess in IPF incidence and mortality in relation to females, as further outlined in Supplementary Fig. 7. Briefly, quantification of diagnoses and deaths in men with and without mLOY shows a clear enrichment in the former. Specifically, we observed that during follow-up time, more than 80% of the male excess of IPF diagnoses and deaths were observed in the group of men with mLOY at study entry.

Since smoking is a replicated risk factor for hematological Y loss as well as IPF, additional exploratory analyses were performed to investigate residual confounding in the associations between mLOY, smoking and IPF. Sensitivity analysis including only smokers (previous and current) first showed that mLOY in blood was associated with risk for IPF mortality also after adjusting for smoking status and dose, estimated by packyears (OR = 1.014, CI = 1.003–1.024, *p* = 0.0117, Supplementary Table 7). The corresponding analysis among never smokers showed a non-significant result in the same direction (Supplementary Table 8). The latter results should be interpreted with caution, given a relatively low number of non-smoking men (*n* = 152) in the dataset being registered with death from IPF in the UKB at the time of data collection. However, the corresponding sensitivity analyses of IPF incidence included larger sample sizes and showed that mLOY increased the risk for IPF diagnosis among both smokers and non-smokers. Specifically, mLOY in blood was associated with increased risk for IPF diagnosis in adjusted logistic regressions including only the current and previously smoking men (OR = 1.008, CI = 1.001–1.014, *p* = 0.0185, Supplementary Table 9). A mediation analysis indicates that about 2% of the increased risk for IPF diagnosis among smokers was mediated by mLOY (*p* < 0.001, Supplementary Table 10). Finally, and of importance, the sensitivity analysis including only the never smoking men replicated the significant association between Y loss in blood cells at study entry and increased risk for IPF diagnosis (OR = 1.02, CI = 1.007–1.031, *p* = 0.0014, Supplementary Table 11).

### Lung function in relation to mLOY

FVC and DLCO are known IPF severity measures^26^. Associations between hematopoietic Y loss and FVC were evaluated in a subset of UKB men with spirometry data passing quality criteria (*n* = 94,154). Initial analyses showed that participants with detectable Y loss in blood cells at study entry displayed significantly reduced FVC in unadjusted analyses (Supplementary Table 12). However, the significance was attenuated when adjusting for confounders (Supplementary Table 13) suggesting that low levels of mLOY do not substantially affect the lung capacity. To investigate the potential effect on lung function in men with high levels of mLOY, we divided the participants into two groups, using 40% mLOY as the cutoff. This allowed us to investigate the lung function of men with a substantial amount of mLOY in blood. In both unadjusted and adjusted analyses, UKB men with >40% mLOY had a significant reduction in FVC compared to other men (Fig. 1e and Supplementary Tables 14 and 15). Since smoking is also a known factor contributing to decreased FVC in men^40^, we evaluated the association between mLOY and FVC in smokers (previous and current) and non-smokers separately (Supplementary Fig. 8). These analyses confirmed that men with >40% mLOY have a significant reduction in FVC compared to other men, regardless of smoking habits (Supplementary Tables 16 and 17).

Next, we evaluated FVC and DLCO in 400 male IPF patients studied in the CleanUP-IPF trial^25^. After QC filtering, 377 patients remained in the analysis and were scored with continuous mLOY. In unadjusted analyses, greater mLOY was associated with increased age (Supplementary Fig. 9) and decreased FVC as well as DLCO (Supplementary Table 18). This result did not, however, remain significant when adjusting for confounders (Supplementary Tables 19 and 20). However, using a categorical mLOY variable representing a high level of mLOY replicated well the results from UKB, showing that patients with mLOY in >40% of blood leukocytes had reduced FVC and DLCO in unadjusted models (Fig. 1f), which remained significant for DLCO (Supplementary Table 21) and marginally significant for FVC in adjusted analyses (Supplementary Table 22).

### IPF patients display higher levels of Y loss in pulmonary leukocytes

**Fig. 2:**
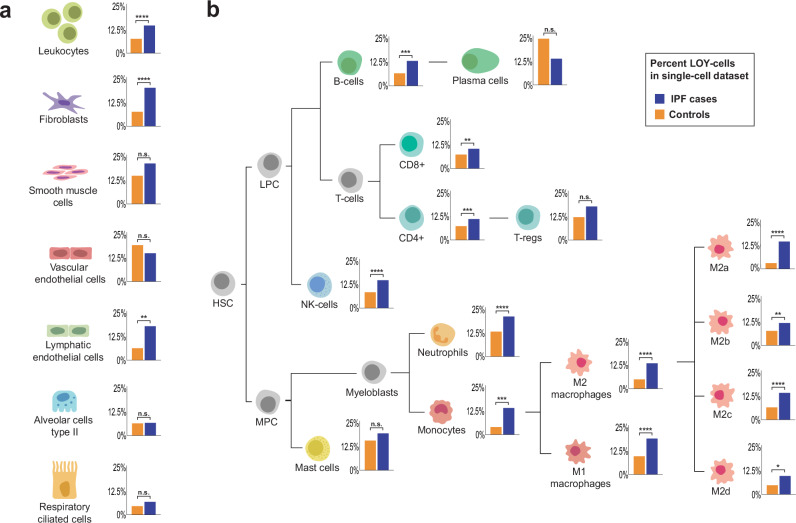
Identified cell types in pulmonary tissues from IPF patients and age-matched controls. Percentage of cells with loss of chromosome Y (LOY) in patients with idiopathic pulmonary fibrosis (IPF) and age-matched healthy controls from the Adams et al. scRNAseq dataset. The bar plot next to each cell type presents the percentage of LOY-cells for that cell population with IPF cases colored in blue and age-matched controls in orange. The *Y*-axis shows the percentage of LOY-cells ranging from 0% to 25%. **a** All cell types detected in the single-cell dataset. **b** Further division of leukocytes into specific cell lineages. Stars mark results from Fisher’s exact tests comparing the number of LOY-cells in IPF cases and age-matched controls, performed for each cell type separately. Significant differences are denoted as: ****p < 0.0001, ***p < 0.001, **p < 0.01, and *p < 0.05. HSC hematopoietic stem cell, LPC lymphoid progenitor cell, MPC myeloid progenitor cell, n.s. not significant.

To characterize mLOY in pulmonary tissues of IPF patients, we first re-analyzed a scRNAseq dataset published by Adams et al.^29^. The processed dataset contained 88,466 single cells from 26 IPF cases and 6 male controls. After clustering and annotation, we could identify hematological, mesenchymal, and epithelial cells in the dataset (Supplementary Fig. 10). We used established methods^8,10,13,41^ to investigate the prevalence of LOY-cells in the identified cell populations in the lung tissue of IPF patients compared with healthy controls (Supplementary Table 23). Across individuals, the percentage of LOY-cells ranged from 3.09-77.33% in IPF cases and 1.57-14.32% in controls (Supplementary Table 24). IPF patients displayed a significantly higher frequency of LOY-cells in leukocytes, fibroblasts, and lymphatic endothelial cells (Fig. 2a and Supplementary Table 25a). Focusing on LOY in leukocytes overall, we observed a two-fold enrichment in IPF patients (Supplementary Table 25a). An increased level of LOY-cells was present in lymphoid as well as myeloid lineages, including NK-cells, B-cells, T-cells, neutrophils, monocytes, and macrophages (Fig. 2b and Supplementary Table 25b). Notably, all lineages derived from myeloblast progenitor cells displayed significantly higher levels of LOY-cells in IPF patients compared with controls (Supplementary Fig. 11 and Supplementary Table 25b) a feature that was most evident in M2a macrophages with a five-fold enrichment in patients (Fisher’s exact test: OR = 5.37, CI = 4.06–7.24, *p* < 0.0001).

### Profibrotic signaling of LOY-leukocytes in IPF patients

**Fig. 3:**
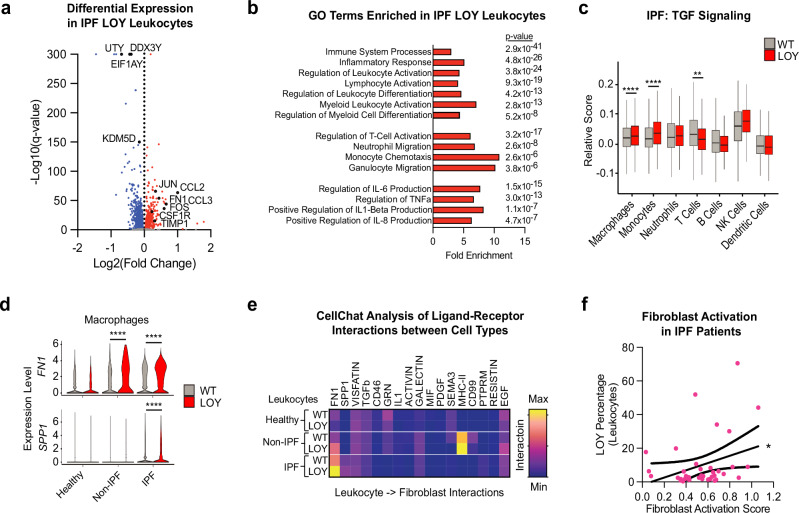
Profibrotic signaling of LOY-leukocytes in men with IPF. Results from single-cell transcriptomics analysis on combined datasets from Morse et al., Reyfman et al., Adams et al., Habermann et al., and de Rooij et al. **a** shows results from the differential gene expression analysis comparing loss of chromosome Y (LOY) cells and wild-type (WT) cells sampled from Idiopathic Pulmonary Fibrosis (IPF) patients. Genes within MSY and genes related to inflammation and fibrosis are labeled in the plot. **b** presents immunological processes enriched in LOY-leukocytes identified using Gene Ontology (GO) terms while **c** shows enrichment of TGF-signaling in specific leukocyte lineages. **d** displays expression levels of the profibrotic genes FN1 and SPP1 in macrophages from healthy, non-IPF (COPD/COVID) and IPF lung dissociates. **e** displays altered ligand-receptor interactions between LOY-leukocytes and other cell types in healthy, non-IPF (COPD/COVID) and IPF, showing all signaling pathways with significant activity as represented by relative interaction strength from minimum activity to maximum activity compared to all interactions. In **f**, the correlation between the percentage of LOY-leukocytes and fibroblast activation in IPF patients is presented. Significant differences are denoted as: ****p < 0.0001, **p < 0.01, *p < 0.05.

Effects from Y loss on genome wide expression of genes and cellular activities were next investigated in a combined dataset composed of five publicly available scRNAseq datasets from lung tissues^27–31^. The pooled and curated dataset consisted of 238,489 single cells originating from 44 IPF patients, 25 non-IPF patients, and 36 control male subjects (Supplementary Fig. 12). LOY-associated DGE was estimated by comparing the expression of genes in LOY-leukocytes with WT-cells sampled from IPF patients. We observed upregulation of specific inflammatory and profibrotic genes such as *FN1, JUN, CCL2, CCL3, FOS, CSF1R*, and *TIMP1* (Fig. 3a). A pathway enrichment analysis of the differentially expressed genes observed in leukocytes from IPF patients suggests an overall enrichment of GO terms associated with immune cell activation processes (Fig. 3b). Furthermore, LOY-macrophages and LOY-monocytes exhibited greater expression of genes related to TGF-signaling (GO:0007179, *p* < 0.0001 by Student *t* test with Bonferroni correction for multiple statistical tests, Fig. 3c). LOY-macrophages also showed higher expression of the profibrotic genes *FN1* and *SPP1* in IPF samples (*p* < 0.0001 by logistic regression likelihood ratio test, Fig. 3d), while other leukocytes had relatively low expression of these genes. Next, changes in cell-to-cell interactions as a consequence of Y loss were explored using CellChat by predicting interactions between LOY-leukocytes with other cell types, using WT-leukocytes as a baseline. This analysis identified strong leukocyte-to-fibroblast interactions and specifically a clear elevation in the profibrotic signaling ligand FN1 in LOY-leukocytes in samples from IPF patients (Fig. 3e). Finally, given the observed enhanced profibrotic signaling of LOY-leukocytes, we investigated if a higher level of Y loss in pulmonary leukocytes was associated with activation of fibroblasts in IPF patients. We found that the percentage of LOY-leukocytes in IPF patients was significantly correlated with increased fibroblast activation score as quantified by average expression of genes enriched in activated pulmonary fibroblasts (*p* < 0.05 by linear regression, Fig. 3f)^38^. We also investigated the age and smoking status of patients and controls included in these analyses to determine whether these variables may have confounding effects on our results. In this combined scRNAseq dataset, there was no correlation between age and mLOY in IPF patients, no difference in smoking status between healthy and IPF patients, and no effect of smoking status on mLOY in different conditions or age-related mLOY (Supplementary Fig. 13). The lack of correlations in age and smoking status suggests that mLOY is the main driver of LOY-enriched pathways and IPF-enriched signaling by LOY-leukocytes from our analyses.

## Discussion

Why men are affected with IPF to a larger extent than women is not well understood, nor is why IPF severity and mortality are higher among men^3^. Previous studies have established that hematopoietic chromosome Y loss, a common and male-specific somatic mutation, is associated with increased risks for the majority of common diseases causing human deaths^6–8,16–18^. Recent results in published^19,20^ as well as pre-print papers^42^ on associations between hematological Y loss and IPF are inconclusive. To further elucidate a potential contribution from Y loss to the male vulnerability for IPF, we performed analyses in epidemiological, clinical and scRNAseq datasets.

Our results from the UKB first established associations between hematological Y loss and increased risk for IPF. Specifically, compared with unaffected men, participants with detectable mLOY in blood cells at study entry displayed a 17.3% increased risk of being diagnosed with IPF (Supplementary Table 6) and a 33.7% increased risk of dying from IPF during almost 15 years follow-up time (Supplementary Table 4). In addition to identifying mLOY in blood cells as a risk factor for IPF, our results in UKB reproduce previously reported risk factors for IPF such as age, smoking and exposure to nanoparticles (Supplementary Tables 3–6). We investigated further the associations between smoking, mLOY and IPF using sensitivity analyses to evaluate potential residual confounding from smoking in our statistical models (Supplementary Tables 7–9 and 11). The retained association between mLOY and risk for IPF in analyses of non-smoking men suggests that Y loss is a risk factor for IPF also in the absence of smoking. Furthermore, mediation analysis performed among the smokers found a moderate but statistically significant mediation effect, suggesting that about 2% of the risk for IPF among smokers was mediated by mLOY (Supplementary Table 10).

It is well known that the prevalence of IPF is higher in men compared to women^3,5^ and here, we note that about 80% of the increased IPF prevalence among men was observed among participants with mLOY (Supplementary Fig. 7). This estimate is not adjusted for confounders such as age and smoking, but suggest that a substantial part of the sex difference in IPF can be explained by mLOY.

A hallmark of IPF severity is reduced lung function, quantified by measures such as FVC and DLCO^26^. Here, we report that UKB participants with high level of Y loss in blood had reduced FVC compared to other men (Fig. 1e and Supplementary Table 15) a decline observed in smokers as well as never smokers (Supplementary Tables 16 and 17). Hence, men with mLOY show signs of reduced lung capacity even if not diagnosed with IPF. Furthermore, reduced capacity of lung gas exchange as estimated by DLCO was observed among IPF patients with a high level of hematopoietic mLOY in the clinical CleanUP-IPF cohort (Fig. 1f and Supplementary Table 21). These results on reduced lung function highlight the clinical relevance of the epidemiological associations between hematological Y loss and IPF observed in UKB.

The mechanisms by which mLOY in blood leukocytes might contribute to various diseases manifesting in other organs are currently being investigated^8,10,13,14,17,39,41^. We have described that hematopoietic mLOY is causally associated with heart failure and reduced lifespan in mouse models^8^. In that study, Y loss in circulating leukocytes led to LOY-macrophages infiltrating the myocardium, triggering cardiac fibrosis by enhanced TGF-β1 signaling. Administration of anti-TGF-β1 antibodies reduced cardiac fibrosis and partly restored cardiac function in LOY-mice. Interestingly, besides myocardial fibrosis, mice with hematopoietic Y loss displayed enhanced fibrosis of lungs and other internal organs^8^. A recent follow-up study identified a chromatin modifying gene located on the Y chromosome, i.e., ubiquitously transcribed tetratricopeptide Y-linked (*Uty*), to be of importance in the profibrotic processes related to complete Y loss and thus to maintain cardiac function^39^. Consistent with this mLOY-mediated profibrotic disease mechanism first discovered in mice, a report of men undergoing transcatheter aortic valve replacement describes gene expression signatures sensitizing circulating LOY-monocytes for TGF-β signaling pathways^17^. It is known that TGF-β1 signaling is a primary factor driving fibrosis in several organs, including lungs^1,2,21,43^. In pulmonary fibrosis it has been suggested that TGF-β1 stimulates *FN1* transcription leading to increased differentiation of fibroblasts to myofibroblasts, which in turn is associated with increased excretion of ECM components such as fibronectin, collagens, and proteoglycans^43–45^. Fibronectin is a fibrogenic cytokine encoded by the *FN1* gene, known to mediate leukocyte-fibroblast communication and to play an important role in the occurrence and progression of fibrosis^43,46^.

Leveraging the power of publicly available human scRNAseq data, we next investigated the role of lung-infiltrating leukocytes with Y loss as a potential activator of this established disease mechanism in men diagnosed with IPF. We first explored single-cell data to characterize the frequency of Y loss in pulmonary leukocytes overall, and observed a two-fold enrichment of LOY-leukocytes in IPF patients compared with controls (Fig. 2a and Supplementary Table 25a). Of note, all myeloid populations displayed significantly higher levels of LOY-cells in IPF patients with up to fivefold enrichment in M2a macrophages (Supplementary Fig. 11 and Supplementary Table 25b), which is a subset of macrophages known to contribute to fibrosis by secretion of TGF-β1^34,36^. To examine the potential functional consequences of this LOY-cell enrichment in the lungs of IPF patients, dysregulated gene expression and affected signaling pathways in pulmonary LOY-leukocytes collected from male IPF patients were investigated (Fig. 3). DGE analyses of pulmonary leukocytes overall showed upregulation of profibrotic genes such as *FN1* in LOY-leukocytes compared to WT-leukocytes (Fig. 3a). Gene set enrichment analysis of the genes dysregulated in LOY-leukocytes highlighted GO terms such as immune system activation and inflammatory processes (Fig. 3b). Remarkably, and in line with previous results from mouse models^8,39^, we found that pulmonary LOY-macrophages and LOY-monocytes derived from IPF patients displayed gene expression signatures consistent with enhanced TGF-signaling pathways (Fig. 3c).

**Fig. 4:**
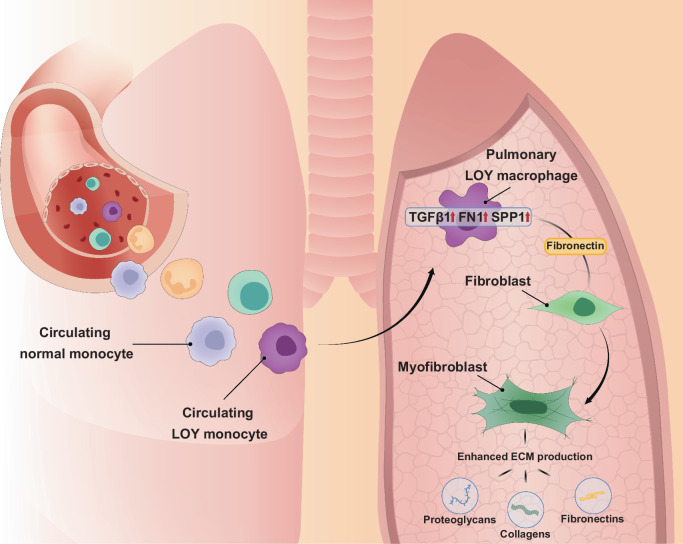
Hypothesized profibrotic disease mechanism by which hematopoietic Y loss in men might exacerbate IPF. We propose an exacerbation of pulmonary fibrosis in male IPF patients with mLOY by activation of established profibrotic pathways. First, circulating LOY-monocytes infiltrate lung tissues and differentiate into pulmonary LOY-macrophages. Next, increased TGF-β1 signaling in LOY-macrophages upregulates *FN1* and *SPP1* expression. Following this, myofibroblast differentiation is stimulated by enhanced FN1-mediated interaction between LOY-macrophages and lung fibroblasts. Myofibroblasts are known for excessive secretion of extracellular matrix (ECM) components such as proteoglycans, collagens, and fibronectins.

Our results from two additional complementary analyses of downstream effects from the dysregulated gene expression of LOY-leukocytes support the hypothesis that Y loss contributes to enhanced fibrosis in male IPF patients. First, we found that *FN1* and *SPP1* were upregulated in LOY-macrophages in IPF patients (Fig. 3d) and that *FN1*-mediated leukocyte-fibroblast interaction was enhanced by the LOY condition within this patient group (Fig. 3e). These results are in line with a recent study employing scRNAseq combined with ligand-receptor interaction analysis in a murine model, showing that macrophages orchestrate fibroblast activation via *Fn1*, *Spp1* and *Sema3* crosstalk^47^. Second, we compared the activation of genes associated with lung fibrosis in fibroblasts of IPF patients in relation to the level of Y loss in their lung resident leukocytes. This analysis showed that male IPF patients with higher levels of Y loss in pulmonary leukocytes displayed enhanced fibroblast activation (Fig. 3f), which in turn has been associated with enhanced lung fibrosis^43^. Taken together, our single-cell analyses suggest that infiltrating LOY-macrophages activate profibrotic signaling pathways in the pulmonary tissue of male IPF patients. Specifically, we propose that enhanced TGF-β signaling of LOY-macrophages stimulates *FN1*-mediated activation of fibroblasts and their differentiation to myofibroblasts, resulting in enhanced ECM production and lung fibrosis (Fig. 4).

A limitation of our study is that the available data did not allow the direct quantification of lung fibrosis in IPF patients in relation to Y loss in circulating and/or tissue resident leukocytes. Further studies including complementary data types such as CT imaging and spatial transcriptomics will be needed for further assessment. Another limitation of the present study is that Y loss in the UKB participants was estimated from whole blood samples, although our scRNAseq data suggest that Y loss in monocytes/macrophages primarily contributes to IPF. Thus, the robustness of our conclusions from whole blood analyses of the UKB dataset would benefit from validation in studies estimating Y loss in circulating monocytes directly. Furthermore, the data from patients and controls in the publicly available scRNAseq datasets were not perfectly matched with regard to age and smoking status. However, as outlined in Supplementary Fig. 13, the lack of correlations with these confounding factors in the analyzed scRNAseq dataset suggests that mLOY is the main driver of our results.

In summary, the profound sex difference in IPF is related to a male-specific genetic risk factor, i.e., the loss of chromosome Y in leukocytes. Our results show that hematological Y loss is associated with increased risk for IPF across ages and smoking habits. We observed that the increased relative risk of IPF mortality (33.7%) among UKB men with mLOY in blood was nearly twice as high as the corresponding risk of IPF diagnosis (17.3%). This suggests that mLOY directly contributes to increasing the severity of IPF etiology, a notion that is supported by the observation of profibrotic signaling mediated by pulmonary LOY-leukocytes in IPF patients. To our knowledge, this is the first report to replicate findings in mice of a mLOY-driven profibrotic disease mechanism in human internal organs. Treatment of IPF is currently limited, but antifibrotic therapies are clinically used to inhibit disease progression. For example, pirfenidone is clinically approved and used to mitigate the cellular effects of TGF-β1 in IPF patients. Based on our findings, we propose that male IPF patients with mLOY might represent a subgroup that would benefit more than other IPF patients from treatment with TGF-β inhibitors.

## Supplementary information

Supplemental material

description of additional supplementary files

Supplementary Data 1

## Data Availability

The numerical data plotted (source data) in Figs. 1, 2, 3, and 4 are provided in Supplementary Data 1. The single-cell RNA sequencing datasets used in this study are publicly available at the GEO browser under accession numbers GSE136831, GSE159585, GSE128033, GSE135893, and GSE122960. UKB and CleanUP-IPF data are available for researchers with permission.
