# Supplementary material for: Mosaic loss of chromosome Y in blood is associated with male susceptibility for idiopathic pulmonary fibrosis
Source: Commun Med (Lond). 2025 Jun 28;5:246. doi: 10.1038/s43856-025-00966-9 (PMC12206232; doi:10.1038/s43856-025-00966-9)
Supplement: Supplementary file 1 — Supplemental material [file 43856_2025_966_MOESM1_ESM.pdf]

Supplementary Information

Mosaic loss of chromosome Y in blood is associated with male susceptibility for Idiopathic Pulmonary Fibrosis

Josefin Bjurling, Nicholas W. Chavkin,† Jonatan Halvardson,† Mark C. Thel, Jonas Mattison, John S. Kim, Ammar Zaghlool, Shwu-Fan Ma, Fernando J. Martinez, Kevin Anstrom, Imre Noth, Kenneth Walsh, Lars A. Forsberg *

†These authors contributed equally to this work
*Corresponding author (lars.forsberg@igp.uu.se)

Content:

Supplementary Figures 1-13
Supplementary Tables 1-25

Supplementary Figures

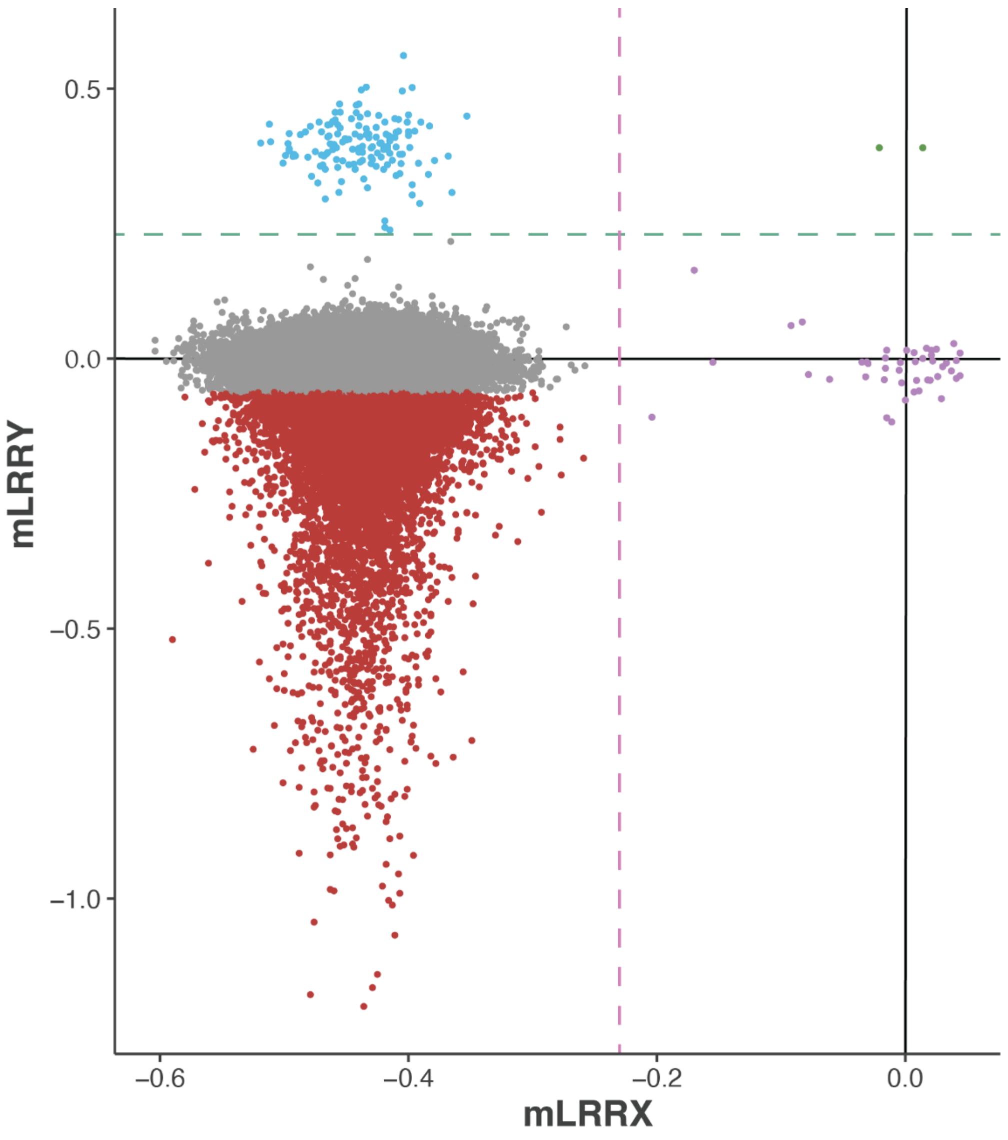

Figure 1. mLRRY and mLRRX values in UK Biobank participants.
Observed sex chromosome aberrations in UK Biobank participants estimated from the calculated medial log R ratio (mLRR) for SNP-array probes on the Y and X chromosome, mLRRY and mLRRX, respectively. An mLRRY value close to zero indicates the presence of one Y chromosome in all cells of a sample and an mLRRX value close to zero indicates the presence of two X chromosomes (black lines). Participants with female as self-reported sex were excluded from the analysis. Furthermore, exclusion was made based on mLRR values as indicated by the pink and green dashed lines. Hence, males with suspected presence of cells with XYY, XXY, or XXYY genotypes (blue, purple, and green dots, respectively) were filtered out.

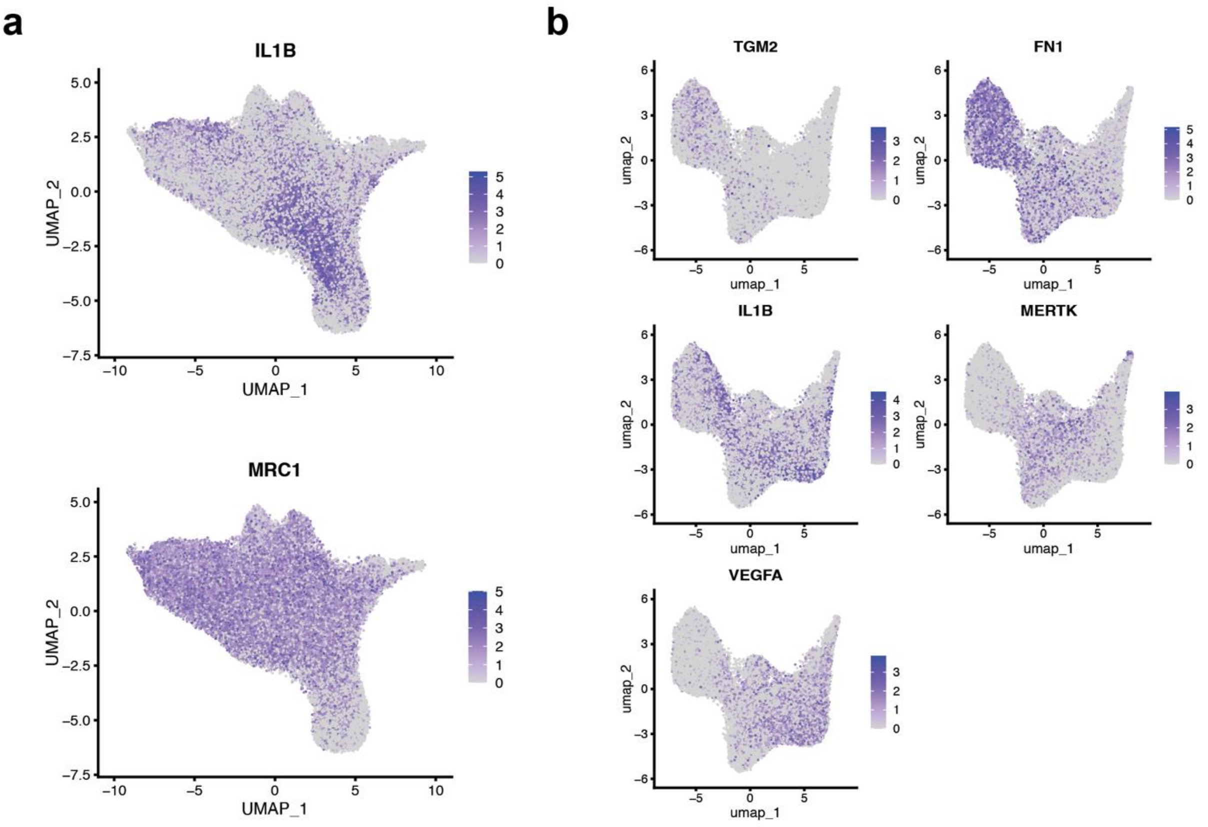

Figure 2. Macrophage subtype markers in Adams *et al.* scRNAseq dataset.
Expression of marker genes to annotate macrophage subtypes in the Adams *et al.* single-cell RNA sequencing (scRNAseq) dataset. Visualised in UMAP plots. **(a)** displays M1 macrophages (*IL1B* gene marker) and M2 macrophages (*MRC1* gene marker) while **(b)** shows gene expression for M2 macrophage subtypes: *TGM2* and *FN1* for M2a, *IL1B* for M2b, *MERTK* for M2c, and high *VEGFA* and low *IL1B* for M2d.

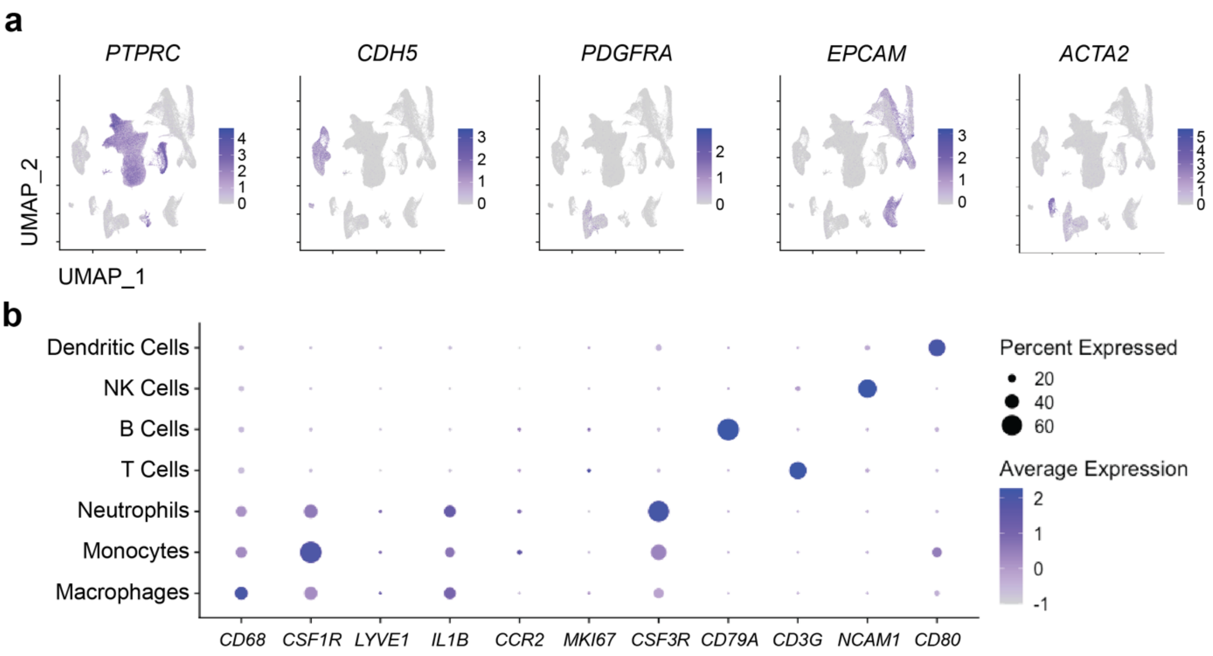

Figure 3. Cell type markers in combined scRNAseq analysis.
Gene expression markers to assign cell types in the combined analysis of single-cell RNA sequencing data from Morse *et al*., Reyfman *et al*., Adams *et al*., Habermann *et al*., and de Rooij *et al*. **(a)** show markers used for the first classification of cells into leukocytes (*PTPRC*), endothelial cells (*CDH5*), fibroblasts (*PDGFRA*), epithelial cells (*EPCAM*), and vascular smooth muscle cells (*ACTA2*). **(b)** Further classification of leukocytes into specific cell types using expression of known marker genes. The size of the circle indicates the percent of expression of marker genes within a cell type while the average expression of marker genes is indicated by the colour intensity.

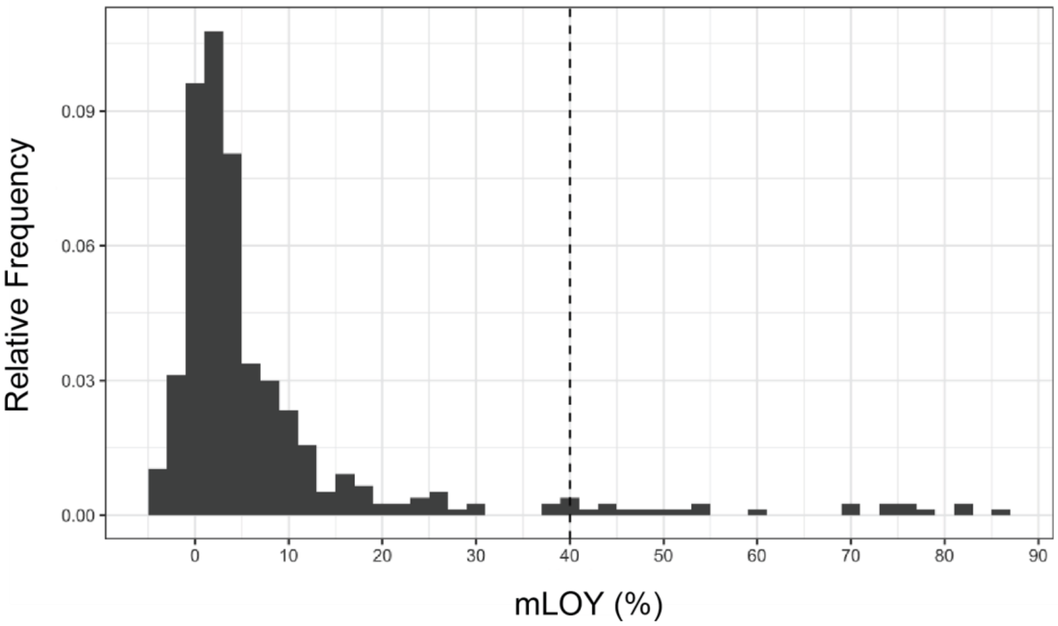

Figure 4. mLOY distribution among male CleanUP-IPF participants.
The histogram displays the relative frequency of men with different levels of mosaic loss of chromosome Y (mLOY) estimated by digital PCR. The dotted line indicates the threshold used to split the cohort into men with more or less than 40% of mLOY.

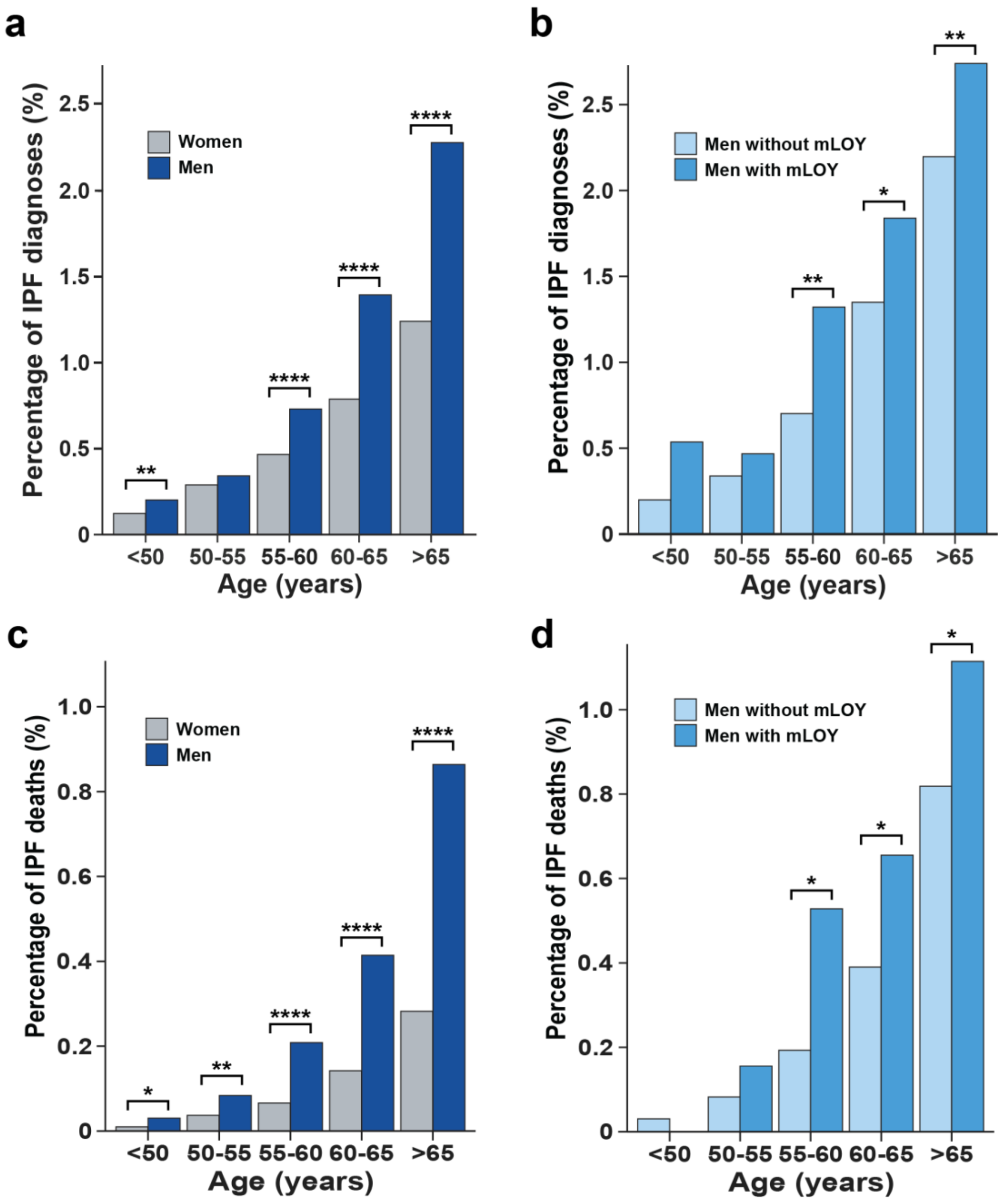

Figure 5. Impact of hematopoietic mLOY on IPF incidence and mortality.
Incidence and mortality from Idiopathic pulmonary fibrosis (IPF) in relation to sex, age, and mLOY status among UK Biobank participants after a median follow-up time of 14.9 years. **(a)** illustrate a male susceptibility for IPF compared with women and an increasing incidence with age. **(b)** show that the frequency of IPF diagnoses was higher among men scored with mLOY compared with other men. **(c)** and **(d)** display corresponding associations with mortality caused by IPF in women and among men with or without mLOY. Stars denote results from Fisher's exact tests comparing the observed number of diagnoses or deaths among women and men (panels A and C) and among men with or without mLOY (panels B and D). Significant differences are denoted as: p <0.0001 ****, p <0.001 ***, p <0.01 **, p <0.05 *. Abbreviations: W = women, M = men, n.s. = not significant.

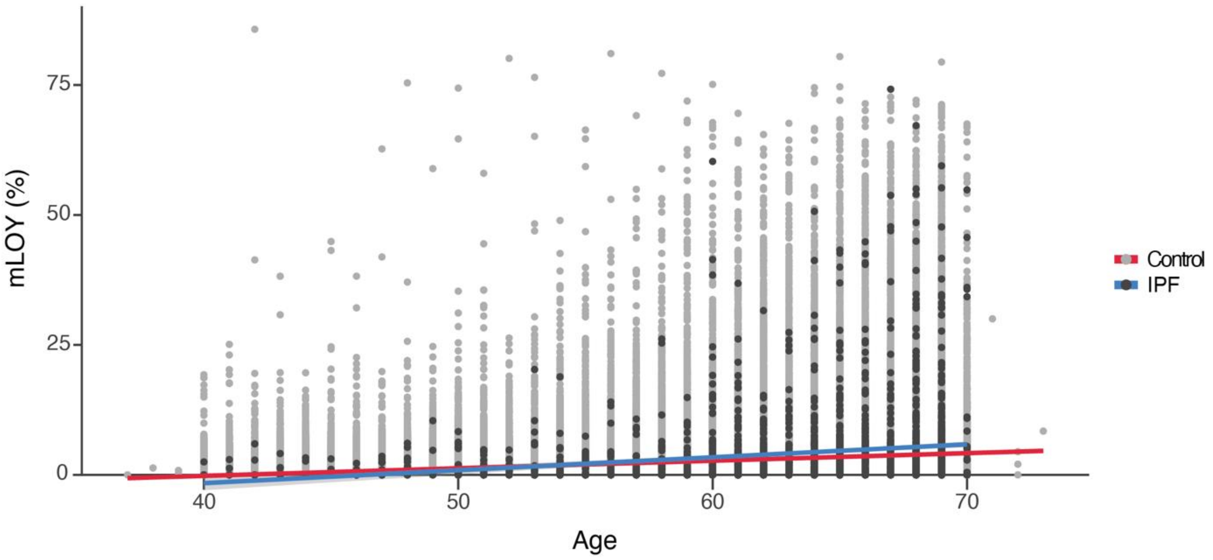

Figure 6. Relationship between age and mLOY in UKB participants.
A scatterplot demonstrating the relationship between age and mLOY percentage in men with idiopathic pulmonary fibrosis (IPF, black dots) and other men (grey dots). A regression line for age and mLOY percentage is plotted in pink for controls and blue for IPF cases.

a

IPF diagnosis	Overall	Women	Men without mLOY	Men with mLOY
Yes	404189	225533	168424	10232
No	2845	1172	1456	217
%	0.7	0.5	0.9	2.1

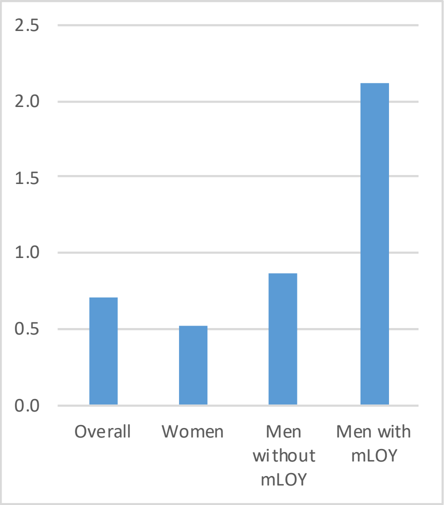

Part of observed sex difference in diagnoses explained by mLOY:

$$\frac{(2.1-0.5)}{(2.1-0.5)+(0.9-0.5)} = \mathbf{0.82}$$

b

IPF death	Overall	Women	Men without mLOY	Men with mLOY
Yes	406303	226494	169441	10368
No	731	211	439	81
%	0.2	0.1	0.3	0.8

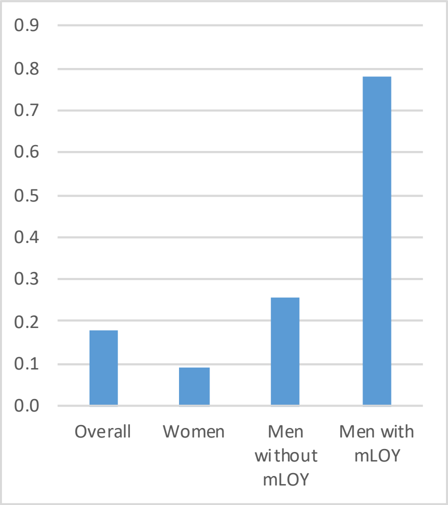

Part of observed sex difference in deaths explained by mLOY:

$$\frac{(0.8-0.1)}{(0.8-0.1)+(0.3-0.1)} = \mathbf{0.81}$$

Figure 7. Estimating the contribution from mLOY to the observed sex differences in incidence and mortality in IPF.

Comparison of the frequencies of IPF diagnoses (A) and IPF deaths (B) in men with and without mLOY to the corresponding frequencies in women in UKB show that mLOY might contribute to about 80% of the observed sex difference in IPF. Hence, these estimates represents the part of the total increased IPF prevalence in men overall (compared with women) that is observed among men with mLOY.

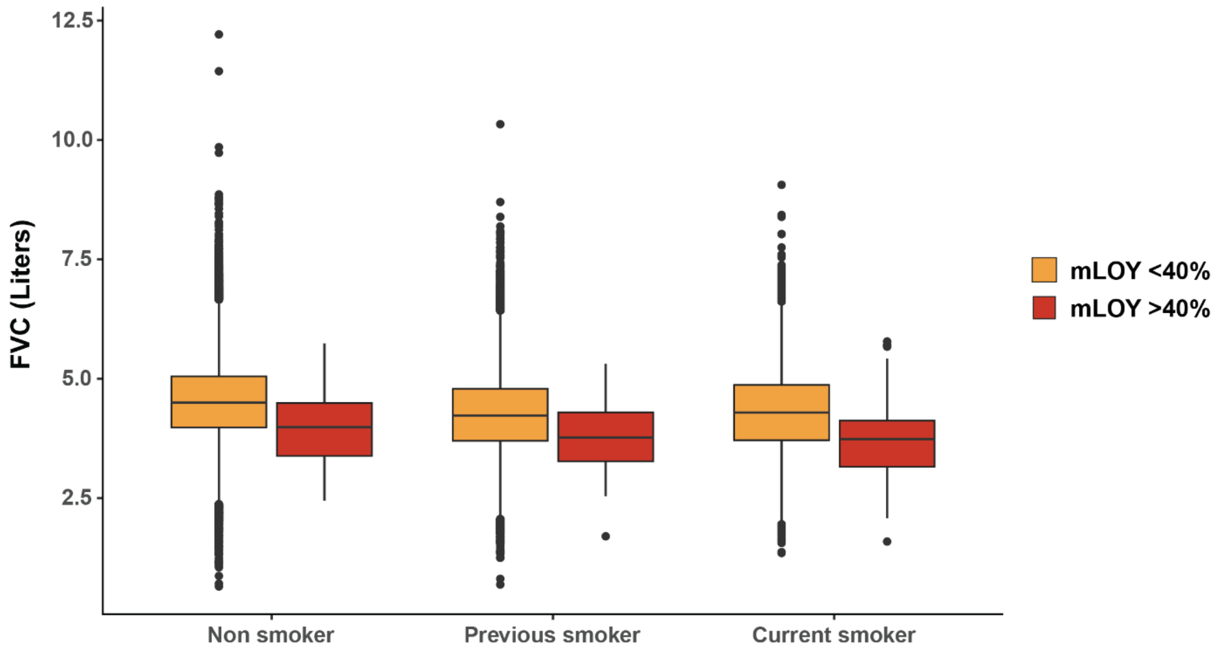

Figure 8. mLOY and FVC based on smoking status in UKB.
Boxplot showing forced vital capacity (FVC) measures in UK Biobank (UKB) men, split based on smoking status. The plot demonstrates differences in FVC measured at baseline between men with more or less than 40% mosaic loss of chromosome Y (mLOY) in each group. Error bars represent the 95% confidence interval.

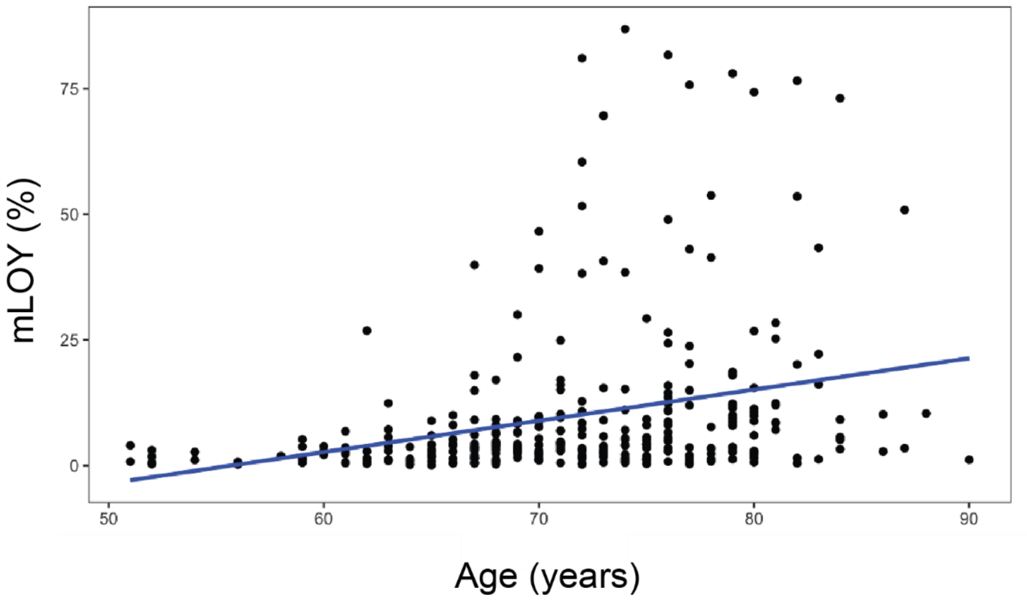

Figure 9. Age and mLOY association in CleanUP-IPF cohort.
Dotplot showing the correlation between mosaic loss of chromosome Y (mLOY) in blood estimated by digital PCR and age in men from the CleanUP-IPF trial.

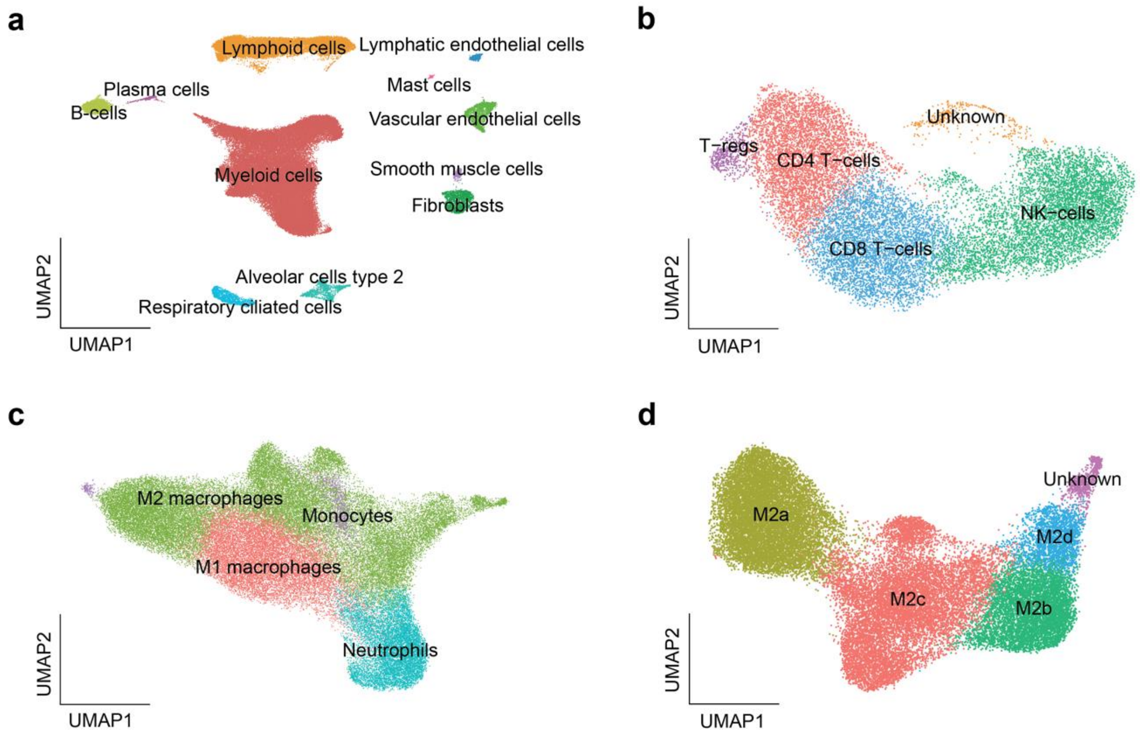

Figure 10. Identified cell types in Adams *et al.* dataset.

Cells types identified in one single-cell RNA sequencing dataset (GSE126831) from 26 Idiopathic Pulmonary Fibrosis and 16 healthy control lungs. Visualised using UMAP. **(a)** All cell types identified in the dataset. The lymphoid and myeloid cell clusters in a were subclustered for identification of specific lymphoid and myeloid lineages, here visualised in panels **(b)** and **(c)**, respectively. **(d)** Further clustering of M2 macrophages in panel c was performed for identification of M2 macrophage subtypes.

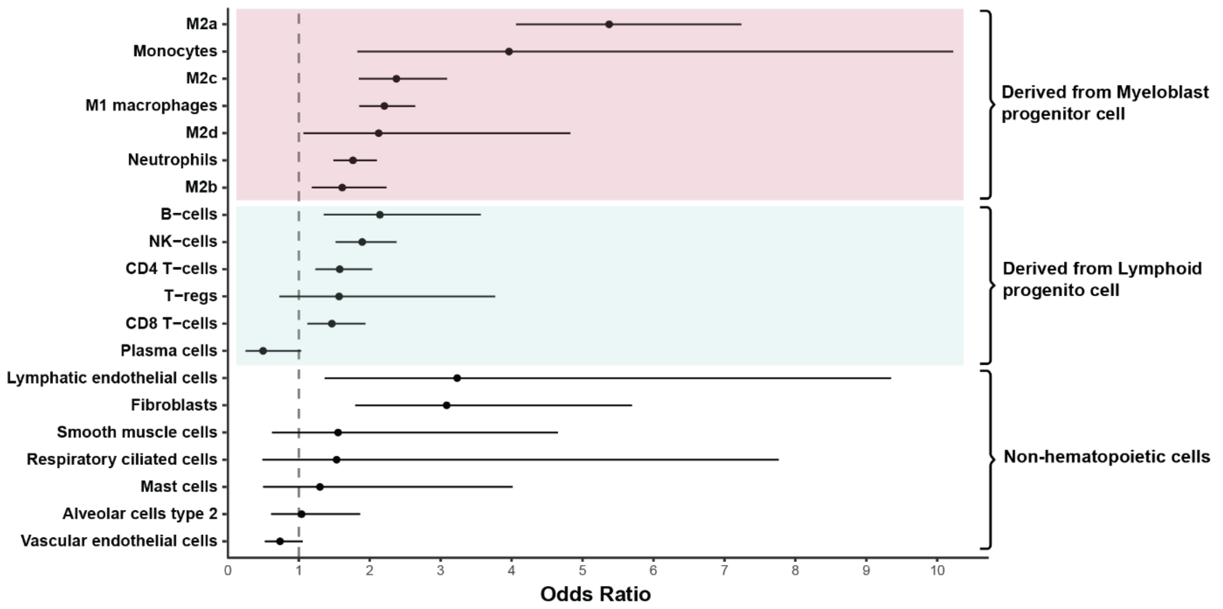

Figure 11. Enrichment of LOY-leukocytes in IPF patients compared with controls. Results from Fisher's exact test comparing the number of loss of chromosome Y (LOY) cells in individuals with Idiopathic pulmonary fibrosis (IPF) and healthy controls in the Adams et al. dataset, performed for each cell type separately. Results visualised with a forest plot showing odds ratios with a 95% confidence interval for each cell type identified in the single-cell RNA sequencing dataset. Cell types derived from myeloid progenitor cells are highlighted in pink while green highlights cell types derived from lymphoid progenitor cells.

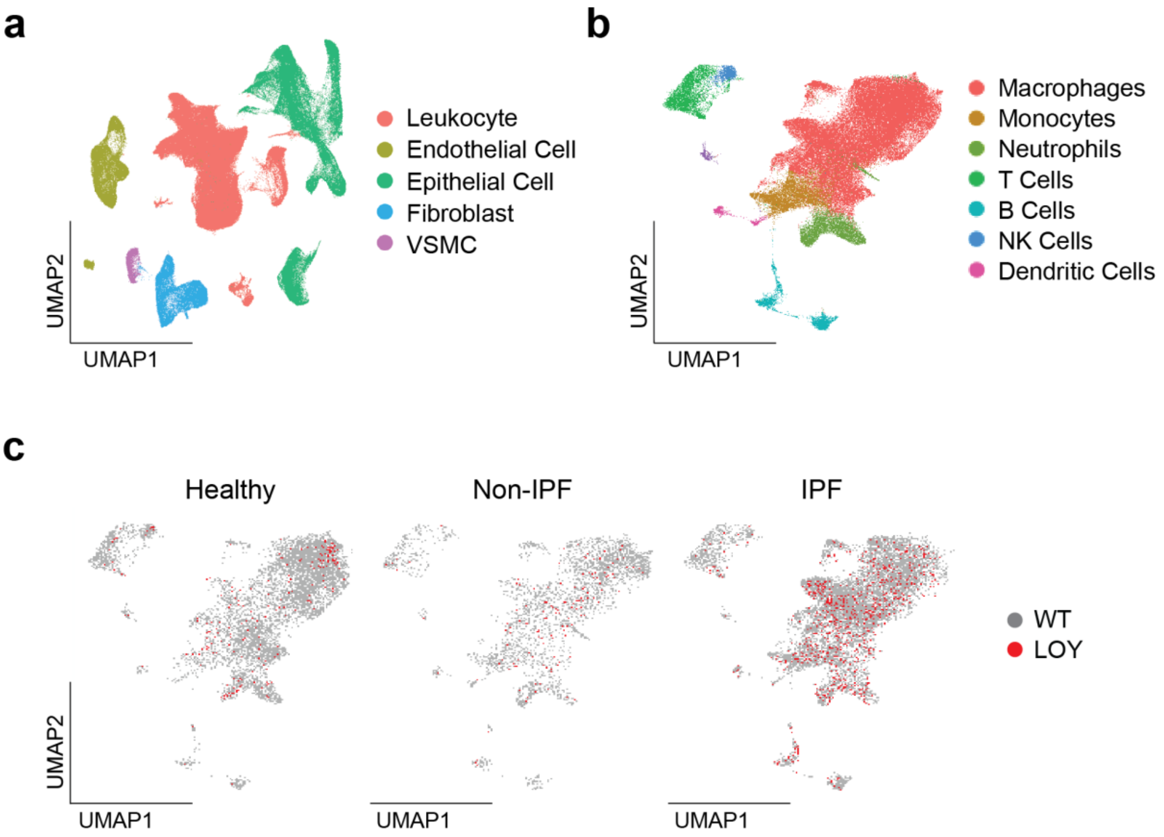

Figure 12. Cell types and LOY-cells identified in pulmonary tissues in five published scRNAseq datasets.

Cell types identified in the single-cell RNA sequencing analysis combining datasets from Morse *et al*., Reyfman *et al*., Adams *et al*., Habermann *et al*., and de Rooij *et al*. Visualised using UMAP. **(a)** show all major cell types identified while **(b)** display leukocyte-specific lineages. **(c)** shows the occurrence of loss of chromosome Y (LOY) cells and wild-type (WT) cells among leukocytes, coloured in red and grey, respectively. Cells from healthy, non-Idiopathic Pulmonary Fibrosis (IPF) (COPD/COVID) and IPF lung dissociates are plotted separately.

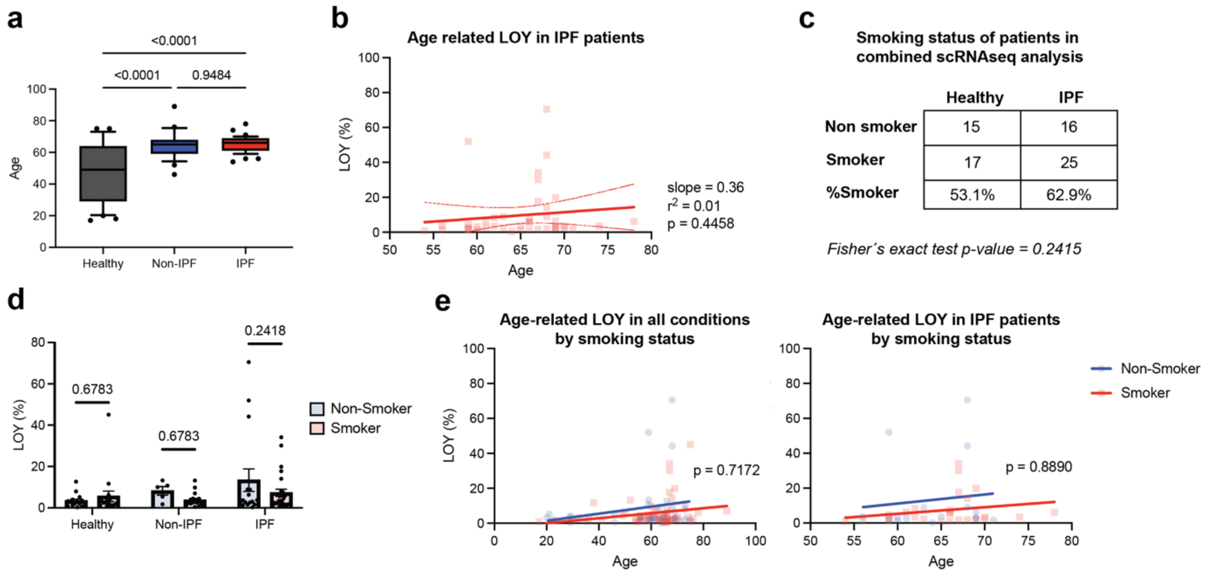

Figure 13. Age and smoking with mLOY in the combined IPF scRNAseq dataset. **(a)** Age range of patients represented in the scRNAseq dataset (one-way ANOVA post-hoc Tukey). **(b)** Correlation between mLOY and age in IPF patient data (linear regression). **(c)** Smoking status of healthy or IPF patients in combined scRNAseq dataset (Fisher's exact test). **(d)** Percent mLOY of patients in different conditions as non-Smokers and smokers (multiple student's t-test). **(e)** Correlation between mLOY and age in all conditions (left) and IPF patients (right) split by smoking status, with statistical comparison of slopes presented (linear regression).

Supplementary Tables

Table 1. IPF incidence in men with or without mLOY in UKB.

Results from Fisher's exact tests comparing the observed number of IPF diagnoses and registered deaths caused by IPF in different age groups in UK Biobank. **(a)** and **(b)** shows comparison between men and women while **(c)** and **(d)** show comparison between men with or without mLOY, set using a threshold of 8.28% mLOY measured in blood. Abbreviations: IPF = Idiopathic Pulmonary Fibrosis, mLOY = mosaic loss of chromosome Y, OR = odds ratio, CI = confidence interval.

a) IPF diagnoses in men compared to women

Age group	OR	95% CI	p-value
<50	1.67	1.23-2.28	0.0008
50-55	1.29	0.96-1.75	0.0865
55-60	1.62	1.35-1.95	< 0.0001
60-65	1.79	1.57-2.03	< 0.0001
>65	1.88	1.65-2.14	< 0.0001

b) IPF deaths in men compared to women

Age group	OR	95% CI	p-value
<50	3.13	1.15-9.86	0.015
50-55	2.62	1.29-5.59	0.0046
55-60	2.68	1.75-4.17	< 0.0001
60-65	2.99	2.26-3.97	< 0.0001
>65	3.09	2.40-4.02	< 0.0001

c) IPF diagnoses in men with mLOY compared to men without mLOY

Age group	OR	95% CI	p-value
<50	3.09	0.37-11.56	0.1409
50-55	1.66	0.34-5.05	0.4339
55-60	2.04	1.26-3.14	0.0025
60-65	1.35	1.04-1.74	0.0208
>65	1.33	1.07-1.63	0.0079

d) IPF deaths in men with mLOY compared to men without mLOY

Age group	OR	95% CI	p-value
<50	-	-	-
50-55	2.01	0.05-12.36	0.4019
55-60	2.76	1.14-5.79	0.0130
60-65	1.66	1.04-2.54	0.0248
>65	1.42	1.01-1.97	0.03452

Table 2. Baseline characteristics for UKB participants.

Baseline characteristics of the confounding factors in the studied UK Biobank cohort. Abbreviations: mLOY = mosaic loss of chromosome Y, IPF = Idiopathic Pulmonary Fibrosis, SD = standard deviation, PC = principal component, PM = particulate matter, FVC = forced vital capacity.

Factor	Overall	Women	Men without mLOY	Men with mLOY
**Number of participants**	407034	226705	169880	10449
**Age**				
Mean (SD)	56.4 (8.09)	56.3 (7.99)	56.1 (8.18)	63.2 (5.16)
Median [Min, Max]	58.0 [38.0, 73.0]	57.0 [39.0, 71.0]	57.0 [38.0, 72.0]	64.0 [40.0, 73.0]
**Smoking**				
Non smoker	262173 (64.4%)	156600 (69.1%)	101387 (59.7%)	4186 (40.1%)
Previous smoker	104780 (25.7%)	50509 (22.3%)	50227 (29.6%)	4044 (38.7%)
Current smoker	40081 (9.8%)	19596 (8.6%)	18266 (10.8%)	2219 (21.2%)
**Packyears**				
Mean (SD)	8.30 (15.8)	6.26 (12.7)	10.3 (18.0)	20.2 (25.1)
Median [Min, Max]	0 [0, 301]	0 [0, 215]	0 [0, 301]	11.5 [0, 220]
**Alcohol consumption**				
Non drinker	20119 (4.9%)	14604 (6.4%)	5244 (3.1%)	271 (2.6%)
Former drinker	14891 (3.7%)	8419 (3.7%)	6044 (3.6%)	428 (4.1%)
Occasional drinker	49135 (12.1%)	35336 (15.6%)	12950 (7.6%)	849 (8.1%)
Drinking one to three times a month	46789 (11.5%)	30058 (13.3%)	15919 (9.4%)	812 (7.8%)
Drinking once or twice a week	105022 (25.8%)	57884 (25.5%)	44669 (26.3%)	2469 (23.6%)
Drinking three or four times a week	91200 (22.4%)	44985 (19.8%)	43761 (25.8%)	2454 (23.5%)
Daily drinker	79878 (19.6%)	35419 (15.6%)	41293 (24.3%)	3166 (30.3%)
**Genetic PC1**				
Mean (SD)	-1.03 (54.6)	-0.280 (56.9)	-1.64 (52.6)	-7.50 (31.1)
Median [Min, Max]	-12.2 [-19.3, 419]	-12.2 [-19.3, 418]	-12.2 [-19.2, 419]	-12.3 [-18.8, 413]
**Genetic PC2**				
Mean (SD)	0.279 (28.2)	0.231 (29.6)	0.282 (26.7)	1.25 (19.3)
Median [Min, Max]	3.65 [-282, 86.1]	3.65 [-282, 84.7]	3.65 [-281, 86.1]	3.66 [-279, 83.0]
**Genetic PC3**				
Mean (SD)	-0.0752 (15.1)	-0.459 (15.4)	0.434 (14.9)	-0.0342 (11.5)
Median [Min, Max]	-1.51 [-143, 98.8]	-1.52 [-143, 98.8]	-1.50 [-141, 97.8]	-1.51 [-139, 93.3]
**Genetic PC4**				
Mean (SD)	0.223 (10.2)	0.225 (10.0)	0.213 (10.6)	0.342 (9.69)
Median [Min, Max]	1.24 [-104, 37.1]	1.23 [-103, 36.4]	1.25 [-104, 37.1]	1.22 [-104, 33.0]
**Genetic PC5**				
Mean (SD)	0.0926 (7.56)	0.0811 (7.56)	0.112 (7.55)	0.0295 (7.80)
Median [Min, Max]	-1.75 [-22.0, 36.2]	-1.72 [-22.0, 33.7]	-1.77 [-19.8, 36.2]	-2.03 [-17.1, 34.6]
**NitrogenDiOxide exposure**				
Mean (SD)	26.6 (7.48)	26.6 (7.43)	26.7 (7.54)	26.5 (7.52)
Median [Min, Max]	26.2 [12.9, 108]	26.2 [12.9, 108]	26.2 [12.9, 108]	26.2 [12.9, 97.1]
**NitrogenOxide Exposure**				
Mean (SD)	44.0 (15.3)	43.8 (15.2)	44.1 (15.5)	43.9 (15.5)
Median [Min, Max]	42.3 [19.7, 266]	42.3 [19.7, 266]	42.3 [19.7, 264]	42.3 [19.7, 241]
**PM10 Exposuure**				
Mean (SD)	16.2 (1.82)	16.2 (1.81)	16.2 (1.83)	16.2 (1.82)
Median [Min, Max]	16.0 [11.8, 30.7]	16.0 [11.8, 30.7]	16.0 [11.8, 30.7]	16.0 [11.8, 30.7]
**PM2.5 Exposure**				
Mean (SD)	9.98 (1.01)	9.97 (1.00)	9.99 (1.02)	9.98 (1.03)
Median [Min, Max]	9.93 [8.17, 21.3]	9.93 [8.17, 21.3]	9.93 [8.17, 21.3]	9.93 [8.17, 19.3]
**PM2.5-PM10 Exposure**				
Mean (SD)	6.40 (0.866)	6.40 (0.863)	6.40 (0.870)	6.39 (0.851)
Median [Min, Max]	6.11 [5.57, 12.8]	6.11 [5.57, 12.8]	6.11 [5.57, 12.8]	6.11 [5.57, 11.2]
**Forced vital capacity**				
Mean (SD)	3.69 (1.06)	3.14 (0.742)	4.42 (0.984)	4.10 (0.931)
Median [Min, Max]	3.56 [0.0200, 32.8]	3.12 [0.0200, 32.8]	4.39 [0.0200, 32.8]	4.08 [0.0400, 32.8]
**Percent LOY**				
Mean (SD)	0.859 (5.90)	-	-0.161 (3.39)	17.4 (11.0)
Median [Min, Max]	0.101 [-29.0, 85.7]	-	-0.159 [-29.0, 8.28]	13.3 [8.28, 85.7]
**IPF diagnose**				
No	404189 (99.3%)	225533 (99.5%)	168424 (99.1%)	10232 (97.9%)
Yes	2845 (0.7%)	1172 (0.5%)	1456 (0.9%)	217 (2.1%)
**IPF death**				
No	406303 (99.8%)	226494 (99.9%)	169441 (99.7%)	10368 (99.2%)
Yes	731 (0.2%)	211 (0.1%)	439 (0.3%)	81 (0.8%)

Table 3. Hematopoietic Y loss and IPF mortality in UKB, continuous mLOY variable. Cox proportional hazards regression for IPF mortality using a continuous mLOY variable. Abbreviations: IPF = Idiopathic Pulmonary Fibrosis, mLOY = mosaic loss of chromosome Y, HR = hazard ratio, CI = confidence interval, PC = principal component, PM = particulate matter.

Factor	HR	95% CI	p-value
**Age**	1.161	1.141-1.182	<0.0001
**Smoking habits**			
Non-smoker (*Reference*)			
Previous smoker	2.050	1.626-2.585	<0.0001
Current smoker	1.850	1.342-2.551	0.0002
**Packyears**	1.010	1.007-1.014	<0.0001
**Alcohol consumption**			
Non-drinker (*Reference*)			
Former drinker	1.262	0.638-2.497	0.5031
Occasional drinker	1.119	0.589-2.125	0.7314
Drinking one to three times a month	1.154	0.608-2.190	0.6613
Drinking once or twice a week	0.921	0.503-1.688	0.7906
Drinking three or four times a week	0.810	0.441-1.490	0.4982
Daily drinker	0.819	0.447-1.500	0.5182
**Genetic components**			
PC1	0.994	0.985-1.002	0.1477
PC2	0.994	0.987-1.000	0.0562
PC3	0.998	0.991-1.005	0.5879
PC4	1.016	1.002-1.029	0.0230
PC5	1.008	0.997-1.020	0.1390
**Environmental exposures**			
Nitrogendioxide	0.983	0.952-1.015	0.3020
Nitrogenoxide	1.001	0.986-1.016	0.9471
PM10	0.949	0.857-1.052	0.3209
PM2.5	1.232	1.060-1.430	0.0064
PM2.5to10	1.064	0.883-1.283	0.5135
**Hematopoietic chromosome Y loss**			
Continuous LOY estimate (%)	1.013	1.003-1.022	0.0088

Table 4. Hematopoietic Y loss and IPF mortality in UKB, categorical LOY variable. Cox proportional hazards regression for IPF mortality using a categorical LOY variable. Defined using a threshold of more or less than 8.28% LOY-cells measured in blood. Abbreviations: IPF = Idiopathic Pulmonary Fibrosis, LOY = loss of chromosome Y, HR = hazard ratio, CI = confidence interval, PC = principal component, PM = particulate matter.

Factor	HR	95% CI	p-value
**Age**	1.162	1.142-1.183	<0.0001
**Smoking habits**			
Non-smoker (*Reference)*			
Previous smoker	2.05	1.626-2.585	<0.0001
Current smoker	1.866	1.354-2.571	0.0001
**Packyears**	1.01	1.007-1.014	<0.0001
**Alcohol consumption**			
Non-drinker (*Reference*)			
Former drinker	1.27	0.642-2.512	0.4922
Occasional drinker	1.122	0.591-2.131	0.725
Drinking one to three times a month	1.157	0.61-2.196	0.6553
Drinking once or twice a week	0.924	0.504-1.693	0.7979
Drinking three or four times a week	0.813	0.442-1.495	0.5055
Daily drinker	0.822	0.449-1.505	0.5257
**Genetic components**			
PC1	0.994	0.985-1.002	0.1461
PC2	0.994	0.987-1	0.0562
PC3	0.998	0.991-1.005	0.5924
PC4	1.016	1.002-1.029	0.0237
PC5	1.008	0.997-1.019	0.1442
**Environmental exposures**			
Nitrogendioxide	0.983	0.952-1.016	0.305
Nitrogenoxide	1	0.986-1.016	0.9525
PM10	0.949	0.857-1.051	0.3178
PM2.5	1.232	1.061-1.431	0.0063
PM2.5to10	1.065	0.884-1.284	0.5088
**Hematopoetic chromosome Y loss**			
Categorical LOY estimate	1.337	1.048-1.706	0.0193

Table 5. Hematopoietic Y loss and IPF incidence in UKB, continuous mLOY variable. Adjusted logistic regression for IPF incidence using a continuous mLOY variable. Abbreviations: IPF = Idiopathic Pulmonary Fibrosis, mLOY = mosaic loss of chromosome Y, OR = odds ratio, CI = confidence interval, PC = principal component, PM = particulate matter.

Factor	OR	95% CI	p-value
**Age**	1.113	1.103-1.123	<0.0001
**Smoking habits**			
Non-smoker (*Reference*)			
Previous smoker	2.011	1.764-2.294	<0.0001
Current smoker	2.011	1.689-2.390	<0.0001
**Packyears**	1.008	1.006-1.010	<0.0001
**Alcohol consumption**			
Non-drinker (*Reference)*			
Former drinker	0.957	0.666-1.393	0.8154
Occasional drinker	0.998	0.719-1.412	0.9899
Drinking one to three times a month	1.010	0.728-1.430	0.9519
Drinking once or twice a week	0.826	0.608-1.146	0.2344
Drinking three or four times a week	0.704	0.517-0.979	0.0306
Daily drinker	0.709	0.523-0.985	0.0334
**Genetic components**			
PC1	0.998	0.996-1.000	0.0538
PC2	0.999	0.997-1.003	0.6880
PC3	1.002	0.997-1.007	0.5277
PC4	1.008	1.003-1.015	0.0047
PC5	1.006	1.000-1.012	0.0555
**Environmental exposures**			
Nitrogendioxide	0.987	0.970-1.005	0.1657
Nitrogenoxide	1.001	0.993-1.009	0.7932
PM10	0.987	0.931-1.048	0.6686
PM2.5	1.195	1.095-1.302	0.0001
PM2.5to10	0.976	0.875-1.088	0.6689
**Hematopoietic chromosome Y loss**			
Continuous LOY estimate (%)	1.010	1.004-1.016	0.0004

Table 6. Hematopoietic Y loss and IPF incidence in UKB, categorical mLOY variable. Adjusted logistic regression for IPF incidence using a categorical LOY variable. Categorical LOY was set with a threshold of more or less than 8.28% LOY-cells measured in blood. Abbreviations: IPF = Idiopathic Pulmonary Fibrosis, LOY = loss of chromosome Y, OR = odds ratio, CI = confidence interval, PC = principal component, PM = particulate matter.

Factor	OR	95% CI	p-value
**Age**	1.114	1.105-1.124	<0.0001
**Smoking habits**			
Non-smoker (*Reference*)			
Previous smoker	2.01	1.763-2.293	<0.0001
Current smoker	2.038	1.712-2.421	<0.0001
**Packyears**	1.008	1.006-1.01	<0.0001
**Alcohol consumption**			
Non-drinker (*Reference*)			
Former drinker	0.959	0.668-1.396	0.8246
Occasional drinker	0.999	0.72-1.414	0.9954
Drinking one to three times a month	1.011	0.729-1.431	0.9482
Drinking once or twice a week	0.827	0.609-1.148	0.2382
Drinking three or four times a week	0.705	0.518-0.981	0.0314
Daily drinker	0.711	0.524-0.988	0.0346
**Genetic components**			
PC1	0.998	0.996-1	0.0512
PC2	0.999	0.997-1.003	0.688
PC3	1.002	0.997-1.007	0.5226
PC4	1.008	1.003-1.015	0.005
PC5	1.006	1-1.012	0.0572
**Environmental exposures**			
Nitrogendioxide	0.987	0.97-1.005	0.1692
Nitrogenoxide	1.001	0.993-1.009	0.8044
PM10	0.987	0.93-1.047	0.6554
PM2.5	1.196	1.096-1.303	0.0001
PM2.5to10	0.977	0.875-1.089	0.681
**Hematopoietic chromosome Y loss**			
Categorical LOY estimate	1.173	1.009-1.358	0.0354

Table 7. Hematopoietic Y loss and IPF mortality among smokers in UKB.
Cox proportional hazards regression for IPF mortality in previous and current smokers using a continuous mLOY variable. Abbreviations: IPF = Idiopathic Pulmonary Fibrosis, mLOY = mosaic loss of chromosome Y, HR = hazard ratio, CI = confidence interval, PC = principal component, PM = particulate matter.

Factor	HR	95% CI	p-value
**Age**	1.143	1.119-1.168	<0.0001
**Smoking habits**			
Previous smoker (*Reference*)			
Current smoker	0.874	0.667-1.146	0.3309
**Packyears**	1.011	1.007-1.014	<0.0001
**Alcohol consumption**			
Non-drinker (*Reference*)			
Former drinker	1.406	0.487-4.056	0.5283
Occasional drinker	1.172	0.413-3.325	0.7654
Drinking one to three times a month	1.137	0.400-3.233	0.8093
Drinking once or twice a week	0.897	0.326-2.469	0.8327
Drinking three or four times a week	0.854	0.310-2.350	0.7595
Daily drinker	0.859	0.314-2.348	0.7676
**Genetic components**			
PC1	0.993	0.982-1.005	0.2569
PC2	0.995	0.986-1.005	0.3451
PC3	0.995	0.984-1.005	0.3244
PC4	1.023	1.002-1.043	0.0287
PC5	1.000	0.987-1.014	0.9741
**Environmental exposures**			
Nitrogendioxide	0.976	0.940-1.013	0.2018
Nitrogenoxide	1.004	0.987-1.021	0.6682
PM10	0.978	0.865-1.106	0.7274
PM2.5	1.281	1.088-1.509	0.0030
PM2.5to10	0.982	0.784-1.230	0.8743
**Hematopoietic chromosome Y loss**			
Continuous LOY estimate (%)	1.014	1.003-1.024	0.0117

Table 8. Hematopoietic Y loss and IPF mortality among never smokers in UKB.
Cox proportional hazards regression for IPF mortality in non-smokers using a continuous mLOY variable. Abbreviations: IPF = Idiopathic Pulmonary Fibrosis, mLOY = mosaic loss of chromosome Y, HR = hazard ratio, PC = principal component, PM = particulate matter.

Factor	HR	95% CI	p-value
**Age**	1.197	1.159-1.237	<0.0001
**Alcohol consumption**			
Non-drinker (*Reference*)			
Former drinker	0.881	0.264-2.938	0.8363
Occasional drinker	1.095	0.460-2.607	0.8372
Drinking one to three times a month	1.287	0.549-3.016	0.5614
Drinking once or twice a week	1.050	0.484-2.275	0.9024
Drinking three or four times a week	0.786	0.355-1.741	0.5527
Daily drinker	0.779	0.348-1.743	0.5429
**Genetic components**			
PC1	0.995	0.983-1.007	0.3750
PC2	0.992	0.983-1.001	0.0861
PC3	1.001	0.992-1.010	0.8019
PC4	1.008	0.991-1.025	0.3677
PC5	1.028	1.007-1.048	0.0073
**Environmental exposures**			
Nitrogendioxide	1.006	0.945-1.071	0.8423
Nitrogenoxide	0.990	0.961-1.021	0.5398
PM10	0.912	0.753-1.104	0.3458
PM2.5	1.066	0.763-1.490	0.7074
PM2.5to10	1.217	0.863-1.716	0.2619
**Hematopoietic chromosome Y loss**			
Continuous LOY estimate (%)	1.011	0.989-1.034	0.3206

Table 9. Hematopoietic Y loss and incidence of IPF among smokers in UKB.
Adjusted logistic regression for IPF incidence in previous and current smokers using a continuous LOY variable. Abbreviations: IPF = Idiopathic Pulmonary Fibrosis, LOY = loss of chromosome Y, OR = odds ratio, CI = confidence interval, PC = principal component, PM = particulate matter.

Factor	OR	95% CI	p-value
**Age**	1.112	1.100-1.1250	<0.0001
**Smoking habits**			
Previous smoker (*Reference*)			
Current smoker	1.019	0.881-1.177	0.7948
**Packyears**	1.008	1.006-1.010	<0.0001
**Alcohol consumption**			
Non-drinker (*Reference*)			
Former drinker	1.105	0.645-2.024	0.7295
Occasional drinker	1.062	0.630-1.920	0.8319
Drinking one to three times a month	0.973	0.575-1.765	0.9241
Drinking once or twice a week	0.857	0.520-1.526	0.5723
Drinking three or four times a week	0.793	0.480-1.412	0.3946
Daily drinker	0.766	0.467-1.359	0.3250
**Genetic components**			
PC1	0.999	0.996-1.001	0.3146
PC2	1.001	0.997-1.005	0.6686
PC3	0.998	0.991-1.005	0.5093
PC4	1.010	1.003-1.019	0.0120
PC5	1.004	0.996-1.011	0.3427
**Environmental exposures**			
Nitrogendioxide	0.980	0.959-1.002	0.0702
Nitrogenoxide	1.002	0.992-1.012	0.7148
PM10	1.009	0.940-1.085	0.8002
PM2.5	1.196	1.078-1.322	0.0060
PM2.5to10	0.952	0.833-1.084	0.4625
**Hematopoietic chromosome Y loss**			
Continuous LOY estimate (%)	1.008	1.001-1.014	0.0185

Table 10. Mediation analysis among smokers in UKB.
Mediation analysis with IPF diagnosis as the dependent variable, packyears as the independent variable, and mosaic loss of chromosome Y (mLOY) as the mediator. Abbreviations: CI = confidence interval.

Effect	Estimate	95% CI	p-value
Total Effect	0.000106	0.0000963 - 0.000114	<0.0001
Average Causal Mediated Effect	0.00000221	0.00000105 - 0.00000335	<0.0001
Average Direct Effect	0.000103	0.0000939 - 0.000112	<0.0001
Proportion mediated effect	0.0211	0.00981 - 0.03	<0.0001

Table 11. Hematopoietic Y loss and incidence of IPF among never smokers in UKB. Adjusted logistic regression for IPF incidence in non-smokers using a continuous LOY variable. Abbreviations: IPF = Idiopathic Pulmonary Fibrosis, LOY = loss of chromosome Y, OR = odds ratio, CI = confidence interval, PC = principal component, PM = particulate matter.

**Factor**	**OR**	**95% CI**	**p-value**
**Age**	1.113	1.098-1.129	<0.0001
**Alcohol consumption**			
Non-drinker (*Reference*)			
Former drinker	0.686	0.345-1.292	0.2600
Occasional drinker	1.008	0.647-1.599	0.9715
Drinking one to three times a month	1.196	0.779-1.878	0.4235
Drinking once or twice a week	0.867	0.588-1.320	0.4885
Drinking three or four times a week	0.627	0.418-0.966	0.0282
Daily drinker	0.683	0.454-1.057	0.0764
**Genetic components**			
PC1	0.998	0.994-1.000	0.0805
PC2	0.998	0.995-1.004	0.4680
PC3	1.004	0.998-1.013	0.2146
PC4	1.005	0.997-1.014	0.2328
PC5	1.011	1.000-1.023	0.0497
**Environmental exposures**			
Nitrogendioxide	1.003	0.971-1.037	0.8465
Nitrogenoxide	1.000	0.985-1.014	0.9571
PM10	0.935	0.843-1.041	0.2131
PM2.5	1.198	1.016-1.408	0.0302
PM2.5to10	1.039	0.852-1.258	0.6969
**Hematopoietic chromosome Y loss**			
Continuous LOY estimate (%)	1.020	1.007-1.031	0.0014

Table 12. Unadjusted model for low level Y loss and FVC in UKB.

Unadjusted generalized linear model for FVC measures in UK Biobank with categorical LOY variable. Categorial LOY was set with a threshold of more or less than 8.28% LOY-cells measured in blood. Abbreviations: FVC = forced vital capacity, mLOY = mosaic loss of chromosome Y, CI = confidence interval.

	Baseline FVC (liters)		
**Factor**	**Estimates**	**95% CI**	**p-value**
Intercept	4.4308	4.4253 - 4.4364	<0.0001
mLOY >8.28% (Y/N)	-0.3447	-0.3681 - -0.3213	<0.0001

Table 13. Adjusted model for low level Y loss and FVC in UKB.

Adjusted generalized linear model for FVC measures in UK Biobank with categorical LOY variable. Categorial LOY was set with a threshold of more or less than 8.28% LOY-cells measured in blood. Abbreviations: FVC = forced vital capacity, CI = confidence interval, BMI = body mass index, PC = principal component, mLOY = mosaic loss of chromosome Y.

	Baseline FVC (liters)		
**Factor**	**Estimates**	**95% CI**	**p-value**
**Age**	-0.0402	-0.0408 - -0.0396	<0.0001
**Smoking habits**			
Non-smoker (*Reference*)			
Previous smoker	0.0777	0.064 - 0.0913	<0.0001
Current smoker	-0.066	-0.0852 - -0.0468	<0.0001
**Packyears**	-0.0064	-0.0068 - -0.006	<0.0001
**BMI**			
18-25	0.4464	0.29 - 0.6028	<0.0001
25-30	0.2738	0.1175 - 0.4301	0.0006
>30	0.0085	-0.1479 - 0.165	0.915
**Genetic components**			
PC1	-0.0018	-0.0033 - -0.0004	0.0141
PC2	0.0026	0.0011 - 0.0041	0.0008
PC3	-0.0029	-0.0049 - -0.0009	0.0039
PC4	-0.0013	-0.0023 - -0.0004	0.0052
PC5	-0.0034	-0.0041 - -0.0028	<0.0001
**Hematopoietic chromosome Y loss**			
mLOY >8.28% (Y/N)	-0.0136	-0.0348 - 0.0076	0.2097

Table 14. Unadjusted model for high level Y loss and FVC in UKB.
Unadjusted generalized linear model for FVC measures in UK Biobank with categorical LOY variable. Categorial LOY was set with a threshold of more or less than 40% LOY-cells measured in blood. Abbreviations: FVC = forced vital capacity, mLOY = mosaic loss of chromosome Y, CI = confidence interval.

	Baseline FVC (liters)		
**Factor**	**Estimates**	**95% CI**	**p-value**
Intercept	4.4135	4.4082 - 4.4189	<0.0001
mLOY >40% (Y/N)	-0.6028	-0.6998 - -0.5059	<0.0001

Table 15. Adjusted model for high level Y loss and FVC in UKB

Adjusted generalized linear model for FVC measures in UK Biobank with categorical LOY variable. Categorial LOY was set with a threshold of more or less than 40% LOY-cells measured in blood. Abbreviations: FVC = forced vital capacity, CI = confidence interval, BMI = body mass index, PC = principal component, mLOY = mosaic loss of chromosome Y.

	Baseline FVC (liters)		
	**Estimates**	**95% CI**	**p-value**
**Age**	-0.0402	-0.0408 - -0.0396	<0.0001
**Smoking habits**			
Non-smoker (*Reference*)			
Previous smoker	0.0777	0.064 - 0.0913	<0.0001
Current smoker	-0.0659	-0.085 - -0.0467	<0.0001
**Packyears**	-0.0064	-0.0068 - -0.006	<0.0001
**BMI**			
18-25	0.4457	0.2893 - 0.6021	<0.0001
25-30	0.2733	0.117 - 0.4295	0.0006
>30	0.0079	-0.1485 - 0.1643	0.9213
**Genetic components**			
PC1	-0.0018	-0.0032 - -0.0004	0.0147
PC2	0.0026	0.0011 - 0.0041	0.0008
PC3	-0.0029	-0.0049 - -0.001	0.0036
PC4	-0.0013	-0.0023 - -0.0004	0.0049
PC5	-0.0034	-0.0041 - -0.0028	<0.0001
**Hematopoetic chromosome Y loss**			
mLOY >40% (Y/N)	-0.1794	-0.2651 - -0.0938	<0.0001

Table 16. mLOY associated with reduced FVC among smokers in UKB.
Adjusted generalized linear model for FVC measures in smoking participants (previous and current) in UK Biobank with categorical LOY variable. Categorial LOY was set with a threshold of more or less than 40% LOY-cells measured in blood. Abbreviations: FVC = forced vital capacity, CI = confidence interval, BMI = body mass index, PC = principal component, mLOY = mosaic loss of chromosome Y.

	**Baseline FVC (liters)**		
	**Estimates**	**95% CI**	**p-value**
**Age**	-0.0427	-0.0437 - -0.0418	<0.0001
**BMI**			
18-25	0.5296	0.3396 - 0.7196	<0.0001
25-30	0.3754	0.1857 - 0.5651	0.0001
>30	0.1196	-0.0704 - 0.3095	0.2173
**Smoking habits**			
Previous smoker (*Reference*)			
Current smoker	-0.1507	-0.1681 - -0.1332	<0.0001
**Packyears**	-0.0063	-0.0066 - -0.0059	<0.0001
**Genetic components**			
PC1	-0.0033	-0.0054 - -0.0011	0.0035
PC2	0.0015	-0.0008 - 0.0038	0.2063
PC3	-0.0048	-0.0078 - -0.0018	0.0016
PC4	-0.0025	-0.004 - -0.001	0.0009
PC5	-0.0033	-0.0043 - -0.0024	<0.0001
**Hematopoietic chromosome Y loss**			
mLOY >40% (Y/N)	-0.14	-0.2379 - -0.042	0.0051

Table 17. mLOY associated with reduced FVC among never smokers in UKB.
Adjusted generalized linear model for FVC measures in non-smoking participants in UK Biobank with categorical LOY variable. Categorial LOY was set with a threshold of more or less than 40% LOY-cells measured in blood. Abbreviations: FVC = forced vital capacity, CI = confidence interval, BMI = body mass index, PC = principal component, mLOY = mosaic loss of chromosome Y.

	Baseline FVC (litres)		
	**Estimates**	**95% CI**	**p-value**
**Age**	-0.0387	-0.0394 - -0.0379	<0.0001
**BMI**			
18-25	0.2559	-0.0164 - 0.5282	0.0655
25-30	0.0733	-0.1989 - 0.3454	0.5978
>30	-0.2027	-0.475 - 0.0697	0.1448
**Genetic components**			
PC1	-0.0007	-0.0027 - 0.0012	0.4543
PC2	0.0035	0.0015 - 0.0055	0.0006
PC3	-0.0015	-0.0042 - 0.0011	0.2607
PC4	-0.0005	-0.0017 - 0.0007	0.4196
PC5	-0.0034	-0.0043 - -0.0025	<0.0001
**Hematopoietic chromosome Y loss**			
mLOY >40% (Y/N)	-0.2548	-0.4277 - -0.0819	0.0039

Table 18. Unadjusted tests for associations between mLOY and the lung function estimators FVC and DLCO in CleanUP-IPF cohort.

Results from unadjusted logistic linear regression. **(a)** Investigating association between mosaic loss of chromosome Y (abbreviated as %LOY) and FVC. **(b)** Results from association test between %LOY and DLCO. Continuous mLOY% was used as the dependent variable. Abbreviations: FVC = forced vital capacity, DLCO = diffusing capacity for carbon monoxide, CI = confidence interval.

a) Baseline FVC

**Predictors**	**Estimates**	**95% CI**	**p-value**
Intercept	2.98	2.89 - 3.07	<0.001
%LOY	-0.01	-0.01 - 0.00	0.02
Observations	377		
R^2 / R^2 adjusted	0.014 / 0.012		

b) Baseline DLCO (actual) (mL/min/mmHg)

**Predictors**	**Estimates**	**95% CI**	**p-value**
Intercept	12.25	11.76 - 12.75	<0.001
%LOY	-0.03	-0.06 - -0.01	0.019
Observations	369		
R^2 / R^2 adjusted	0.015 / 0.012		

Table 19. Multiple linear regression for FVC with continuous LOY in CleanUP-IPF. Results from multiple linear regression for FVC measurements with continuous loss of chromosome Y (abbreviated as % LOY) as the dependent variable. Baseline confounders used for adjustment are shown in the table. Abbreviations: FVC = forced vital capacity, CI = confidence interval

	Baseline FVC			Baseline FVC % Predicted		
**Predictors**	**Estimates**	**95% CI**	**p-value**	**Estimates**	**95% CI**	**p-value**
Intercept	3.81	3.00 - 4.61	<0.001	36.25	17.74 - 54.76	<0.001
%LOY	-0.00	-0.01 - 0.00	0.163	-0.09	-0.22 - 0.03	0.148
Age (Years)	-0.01	-0.02 - -0.00	0.025	0.46	0.20 - 0.73	0.001
Smoking Status	0.19	0.03 - 0.36	0.024	4.59	0.76 - 8.43	0.019
Coronary artery disease	-0.13	-0.31 - 0.05	0.145	-4.16	-8.22 - -0.11	0.044
Congestive heart failure	0.12	-0.29 - 0.53	0.552	0.15	-9.18 - 9.47	0.975
Observations	377			370		
R^2 / R^2 adjusted	0.046 / 0.033			0.052 / 0.039		

Table 20. Multiple linear regression for DLCO with continuous LOY in CleanUP-IPF. Results from multiple linear regression for DLCO measurements with continuous loss of chromosome Y (abbreviated as % LOY) as the dependent variable. Baseline confounders used for adjustment are shown in the table. Abbreviations: DLCO = diffusing capacity for carbon monoxide, CI = confidence interval.

	Baseline DLCO (actual) (mL/min/mmHg)			Baseline DLCO % Predicted (Analysis)		
**Predictors**	**Estimates**	**95% CI**	**p-value**	**Estimates**	**95% CI**	**p-value**
Intercept	24.36	20.09 - 28.62	<0.001	53.73	40.34 - 67.12	<0.001
%LOY	-0.01	-0.04 - 0.02	0.39	-0.04	-0.13 - 0.05	0.435
Age (Years)	-0.17	-0.23 - -0.11	<0.001	-0.19	-0.38 - -0.00	0.049
Smoking Status	-0.13	-1.02 - 0.75	0.772	-0.13	-2.91 - 2.66	0.927
Coronary artery disease	-0.47	-1.41 - 0.46	0.322	-1.50	-4.47 - 1.46	0.319
Congestive heart failure	-2.5	-4.65 - -0.35	0.023	-9.07	-15.82 - -2.33	0.009
Observations	369			364		
R^2 / R^2 adjusted	0.116 / 0.104			0.044 / 0.031		

Table 21. Multiple linear regression for DLCO with categorial LOY in CleanUP-IPF. Adjusted multiple linear regression for DLCO measurements with categorial LOY as the dependent variable. Categorial LOY was set with a threshold of more or less than 40% LOY-cells estimated by digital PCR. Abbreviations: DLCO = diffusing capacity for carbon monoxide, LOY = loss of chromosome Y, CI = confidence interval.

	Baseline DLCO % Predicted (Analysis)			Baseline DLCO (actual) (mL/min/mmHg)		
**Predictors**	**Estimates**	**95% CI**	**p-value**	**Estimates**	**95% CI**	**p-value**
Intercept	52.73	39.54 - 65.92	<0.001	24.04	19.84 - 28.23	<0.001
LOY > 40 %	-6.19	-12.08 - -0.29	0.04	-2.1	-3.98 - -0.22	0.029
Age (Years)	-0.18	-0.36 - 0.01	0.061	-0.16	-0.22 - -0.10	<0.001
Smoking Status	-0.12	-2.88 - 2.64	0.931	-0.13	-1.00 - 0.75	0.777
Coronary artery disease	-1.32	-4.28 - 1.63	0.379	-0.41	-1.35 - 0.52	0.383
Congestive heart failure	-9.07	-15.78 - -2.36	0.008	-2.5	-4.64 - -0.36	0.022
Observations	364			369		
R^2 / R^2 adjusted	0.054 / 0.041			0.126 / 0.114		

Table 22. Multiple linear regression for FVC with categorial LOY in CleanUP-IPF. Adjusted multiple linear regression for FVC measurements with categorial LOY as the dependent variable. Categorial LOY was set with a threshold of more or less than 40% LOY-cells estimated by digital PCR. Abbreviations: FVC = forced vital capacity, LOY = loss of chromosome Y, CI = confidence interval.

	Baseline FVC			Baseline FVC % Predicted		
**Predictors**	**Estimates**	**95% CI**	**p-value**	**Estimates**	**95% CI**	**p-value**
Intercept	3.81	3.01 - 4.61	<0.001	36.55	18.26 - 54.83	<0.001
LOY > 40 %	-0.35	-0.71 - 0.01	0.058	-7.58	-15.76 - 0.61	0.07
Age (Years)	-0.01	-0.02 - -0.00	0.019	0.45	0.20 - 0.71	0.001
Smoking Status	0.2	0.03 - 0.36	0.02	4.73	0.92 - 8.54	0.015
Coronary artery disease	-0.12	-0.30 - 0.05	0.17	-3.99	-8.04 - 0.06	0.054
Congestive heart failure	0.13	-0.28 - 0.54	0.536	0.26	-9.05 - 9.57	0.956
Observations	377			370		
R^2 / R^2 adjusted	0.050 / 0.038			0.055 / 0.042		

Table 23. Percentage LOY in single-cell dataset from Adams *et al*.

The occurrence of LOY-cells in the Adams *et al.* dataset, presented as the percentage of the total number of cells. The percentage of LOY-cells is calculated for the entire dataset (total), as well as for men with IPF and healthy men (control) separately. **(a)** Percentage LOY-cells for all identified cell types. **(b)** LOY-cells in leukocyte subsets. Abbreviations: LOY = loss of chromosome Y, IPF = Idiopathic Pulmonary Fibrosis, NR = number.

a) All identified cell types

	IPF		Control		Total	
**Cell type**	**LOY cells (%)**	**Cells (NR)**	**LOY cells (%)**	**Cells (NR)**	**LOY cells (%)**	**Cells (NR)**
Leukocytes	15.0	50052	7.8	10792	13.7	60844
Alveolar cells type 2	6.72	1355	6.47	278	6.67	1633
Fibroblasts	20.45	1804	7.69	195	19.21	1999
Lymphatic endothelial cells	18.09	470	6.38	94	16.13	564
Mast cells	19.59	245	15.79	38	19.08	283
Respiratory ciliated cells	6.91	1520	4.62	65	6.81	1585
Smooth muscle cells	21.53	483	15	40	21.03	523
Vascular endothelial cells	15.02	1778	19.37	253	15.56	2031

b) Leukocyte subsets

	IPF		Control		Total	
**Cell type**	**LOY cells (%)**	**Cells (NR)**	**LOY cells (%)**	**Cells (NR)**	**LOY cells (%)**	**Cells (NR)**
B-cells	13.24	2560	6.65	316	12.52	2876
Plasma cells	13.93	359	24.59	61	15.48	420
NK-cells	14.86	4435	8.44	1221	13.47	5656
CD4 T-cells	11.1	3432	7.34	1186	10.13	4618
CD8 T-cells	10.49	3336	7.4	973	9.79	4309
T-regs	17.86	364	12.16	74	16.89	438
Neutrophils	21.29	5129	13.29	1384	19.59	6513
Monocytes	14.18	973	4	175	12.63	1148
M1 macrophages	19.14	8424	9.69	1621	17.61	10045
M2 macrophages	13.51	21040	4.95	3781	12.2	24821
M2a	14.37	7555	3.03	1783	12.21	9338
M2b	11.87	3624	7.7	649	11.23	4273
M2c	14.27	7790	6.55	1084	13.33	8874
M2d	9.81	1539	4.86	185	9.28	1724

Table 24. Lung donor characteristics in Adams *et al.*

Characteristics for lung donors in the single-cell RNA sequencing dataset from Adams *et al.* Abbreviations: IPF = Idiopathic Pulmonary Fibrosis, LOY = loss of chromosome Y, NR = number.

Subject ID	Age	Ethnicity	Ever Smoker	Disease Group	LOY (%)	Cells (NR)	Included in statistical analysis
034I	54	white	Yes	IPF	6.93	2452	Yes
138I	56	white	Yes	IPF	4.52	1967	Yes
228I	56	white	No	IPF	12.61	1760	Yes
041I	59	white	No	IPF	4.05	1829	Yes
135I	59	white	Yes	IPF	4.61	3430	Yes
222I	59	white	No	IPF	53.24	4844	Yes
053I	60	white	Yes	IPF	5.5	1963	Yes
29I	61	white	No	IPF	5.71	315	Yes
051I	62	other	Yes	IPF	12.03	2909	Yes
166I	63	white	Yes	IPF	9.59	4079	Yes
47I	64	white	No	IPF	3.09	939	Yes
025I	65	white	Yes	IPF	4.84	1095	Yes
49I	66	white	Yes	IPF	5.22	2699	Yes
022I	67	white	Yes	IPF	7.39	2721	Yes
145I	67	white	Yes	IPF	20.82	3089	Yes
063I	68	white	No	IPF	77.33	1853	Yes
158I	68	white	Yes	IPF	6.96	4469	Yes
210I	68	white	Yes	IPF	5.66	990	Yes
021I	69	white	No	IPF	9.65	1316	Yes
177I	69	white	Yes	IPF	25.25	2709	Yes
214I	69	white	Yes	IPF	6.79	383	Yes
040I	70	white	Yes	IPF	6.84	2075	Yes
179I	70	white	Yes	IPF	7.98	2756	Yes
212I	71	white	No	IPF	5.4	1926	Yes
123I	74	white	Yes	IPF	7.65	1020	Yes
010I	78	white	Yes	IPF	9.02	2460	Yes
081C	20	white	No	Control	7.93	227	No
001C	22	white	No	Control	5.06	1027	No
208C	23	white	No	Control	5.82	825	No
092C	29	latino	No	Control	1.57	2491	No
218C	29	white	No	Control	12.89	3537	No
484C	31	white	Yes	Control	6.27	542	No
226C	32	white	Yes	Control	12.38	8674	No
483C	35	white	No	Control	4.01	748	No
084C	46	black	No	Control	5.79	190	No
034C	49	asian	Yes	Control	8.29	350	No
244C	50	white	Yes	Control	12.73	110	Yes
137C	54	white	No	Control	3.46	578	Yes
465C	56	white	No	Control	4.72	1736	Yes
388C	61	white	No	Control	14.32	1292	Yes
160C	64	white	No	Control	4.72	2286	Yes
222C	65	white	Yes	Control	9.23	5805	Yes

Table 25. Enrichment of cells with Y loss in IPF patients.

Results from Fisher's exact test comparing the number of LOY-cells in IPF cases and controls in the Adams *et al*. dataset. Test performed for **(a)** all identified cell types in the dataset individually and **(b)** all leukocyte subsets. Abbreviations: OR = Odds ratio, CI = confidence interval.

a) All identified cell types

Cell type	OR	95% CI	p-value
Alveolar cells type 2	1.04	0.61-1.87	1
Fibroblasts	3.08	1.79-5.7	<0.0001
Leukocytes	2.09	1.94-2.25	<0.0001
Lymphatic endothelial cells	3.23	1.36-9.35	0.0034
Respiratory ciliated cells	1.53	0.49-7.77	0.6197
Smooth muscle cells	1.55	0.62-4.65	0.4212
Vascular endothelial cells	0.74	0.52-1.05	0.0783

b) Leukocyte subsets

Cell type	OR	95% CI	p-value
B-cells	2.14	1.35-3.57	<0.001
Plasma cells	0.5	0.25-1.03	0.0532
NK-cells	1.89	1.52-2.38	<0.0001
CD4 T-cells	1.58	1.23-2.04	<0.001
CD8 T-cells	1.47	1.12-1.94	0.0039
T-regs	1.57	0.73-3.77	0.3068
Mast cells	1.3	0.5-4.02	0.6635
Neutrophils	1.76	1.49-2.1	<0.0001
Monocytes	3.96	1.83-10.22	<0.001
M1 macrophages	2.21	1.85-2.64	<0.0001
M2 macrophages	3	2.58-3.51	<0.0001
M2a	5.37	4.06-7.24	<0.0001
M2b	1.61	1.18-2.24	0.0015
M2c	2.38	1.85-3.09	<0.0001
M2d	2.13	1.06-4.83	0.0308
