# Supplementary material for: Mosaic loss of chromosome Y in blood is associated with male susceptibility for idiopathic pulmonary fibrosis
Source: Commun Med (Lond). 2025 Jun 28;5:246. doi: 10.1038/s43856-025-00966-9 (PMC12206232; doi:10.1038/s43856-025-00966-9)
Supplement: Supplementary file 2 — description of additional supplementary files [file 43856_2025_966_MOESM2_ESM.pdf]

Description of Additional Supplementary Files

File name: Supplementary Data 1

Description: Source data used for generating all main figures.
